# Real-world effectiveness and impact of the 4CMenB vaccine against serogroup B invasive meningococcal disease: a systematic review and meta-analysis

**DOI:** 10.1038/s41541-026-01511-y

**Published:** 2026-07-06

**Authors:** Pavo Marijic, Katarzyna Jamróz-Dolińska, Wojciech Margas, Lucian Gaianu, Gaurav Mathur, Piotr Wojciechowski, Thatiana Pinto, Tia Vincent, Helen Petousis-Harris, Terry Nolan, Federico Martinón-Torres, Lee H. Harrison, Zeki Kocaata

**Affiliations:** 1 GSK, Munich, Germany; 2 Clever-Access, Kraków, Poland; 3 GSK, London, UK; 4 GSK, Rockville, MD USA; 5 GSK, Wavre, Belgium; 6 University of Auckland, Auckland, New Zealand; 7 University of Melbourne, Melbourne, VIC Australia; 8 Translational Pediatrics and Infectious Diseases, Hospital Clínico Universitario de Santiago (SERGAS) and University of Santiago de Compostela (USC), Santiago de Compostela, Spain; 9 Genetics, Vaccines and Pediatric Infectious Diseases Research Group (GENVIP), Instituto de Investigación Sanitaria de Santiago de Compostela (IDIS), Santiago de Compostela, Spain; 10 Centro de Investigación Biomédica en Red de Enfermedades Respiratorias (CIBERES), Instituto de Salud Carlos III, Madrid, Spain; 11 WHO Collaborating Centre for Vaccine Safety, Santiago de Compostela, Spain; 12 Center for Genomic Epidemiology, University of Pittsburgh School of Medicine, Pittsburgh, PA USA

**Keywords:** Diseases, Health care, Immunology, Medical research, Microbiology

## Abstract

4CMenB vaccine is authorized for protection against serogroup B invasive meningococcal disease (IMD). This study synthesized real-world evidence (RWE) data on effectiveness and impact of 4CMenB vaccine. A systematic search identified RWE on vaccine effectiveness (VE) and vaccine impact of 4CMenB in infants, children and adolescents. A meta-analysis was conducted of 4CMenB VE against serogroup B-IMD in infants and children. The primary meta-analysis used a random-effects model on data from five studies from five countries reporting VE in fully vaccinated infants and children, and estimated pooled VE at 79.7% (95% confidence interval 70.4, 86.1). In sensitivity analyzes, inclusion/exclusion of studies from the primary analysis did not materially change the results. Age-specific data in adolescents were summarized qualitatively. Data identified from Australia reported high effectiveness and impact in adolescents. This meta-analysis provides evidence of high 4CMenB VE against serogroup B-IMD in fully vaccinated infants and children across different geographic regions. Clinical trial registration: N/A.

## Introduction

Invasive meningococcal disease (IMD) is a serious acute infection caused by the bacterium *Neisseria meningitidis*. It most commonly presents as meningitis and/or septicemia, can progress rapidly and may be fatal within hours^1^. In a meta-analysis including data from 40 studies, case-fatality rates for IMD ranged from 4.1% to 20.0%, with a pooled case-fatality rate of 8.3%^2^. Up to 20% of survivors may experience long-term sequelae such as neurological impairment, hearing loss, or amputation^3^. In a case-control study among survivors of serogroup B IMD in the UK, 9% of survivors experienced major disabling deficits, and over one-third had one or more deficits in physical, cognitive or psychological functioning^4^. In countries with available epidemiological data, IMD incidence is typically highest in infants and young children, with a second peak observed in adolescents and young adults in the United States (US), Europe and Australasia^1,5,6^. The high incidence in adolescents and young adults may be affected by social and behavioral factors such as living in crowded accommodation, smoking, attending social gatherings, and exposure to circulating strains not previously encountered^7^. Also, classic and contemporary sero-epidemiological studies show that naturally acquired meningococcal antibodies are lowest in early childhood (particularly after loss of maternal antibody), rise with increasing age through later childhood and adolescence, and then plateau or decline in older age groups, with patterns varying by serogroup and setting^8–13^.

*N. meningitidis* is classified into groups based on variations in the polysaccharide capsule, called “capsular groups” or “serogroups”. In this paper, the term “serogroup” is used. Six serogroups (A, B, C, W, X and Y) are responsible for most IMD cases^6^. Across Europe, North America and Australasia, serogroup B is the predominant serogroup in IMD, and serogroups C and W account for a substantial proportion of cases in Africa, Latin America and parts of Asia^6^. IMD incidence and serogroup distribution are complex and dynamic, varying by geographic region and over time, with disease trends influenced by natural fluctuations, the effects of public health interventions and behavioral and environmental factors^6^. Vaccines are available against all major disease-causing serogroups, with MenACWY and MenB vaccines implemented in many countries across the world^6,14^, and a MenACWXY vaccine (*MenFive*, Serum Institute of India) has been developed and is recommended for use in all countries in the African meningitis belt^15^. The first broad-spectrum protein-based MenB vaccine to be developed was 4CMenB (*Bexsero*, GSK), which was licensed in 2013 for use in individuals aged 2 months or older in Europe, followed a year later by MenB-FHpb (*Trumenba*, Pfizer) for use in individuals aged 10–25 years^16^. The 4CMenB vaccine is an outer membrane vesicle (OMV)-based vaccine, and other OMV-based vaccines have also been developed against MenB and other pathogens such as *Hemophilus influenzae* type b^17^. In some geographical regions, including North America, Europe and Oceania, 4CMenB is the only MenB vaccine authorized for use in infants and children. Real-world data on 4CMenB from over 10 years of use have supported the clinical efficacy and safety data obtained from early trials^16^. 4CMenB vaccination is recommended and reimbursed or fully funded in national and regional immunization programs in several countries globally^16^, and is mandatory for infants in France following a recommendation from the Haute Autorité de Santé in March 2024^18^.

Real-world evidence (RWE) data are available for 4CMenB vaccine effectiveness (VE) and vaccine impact (VI) from the UK, Italy, Spain, Portugal, the US, Canada and Australia^16^. However, uncertainty remains over the duration of protection, especially in the population most at risk from IMD, infants and young children. In addition, heterogeneity in study designs, populations, vaccine schedules, and follow-up periods makes interpretation of the data challenging. A previous systematic literature review (SLR) of real-world studies on 4CMenB VE and VI identified five studies^19^. Diversity in vaccine schedule, populations and analysis methods, reflecting variations in vaccine strategies in the study settings, meant that quantitative pooling methods could not be applied^19^. Since the publication of this SLR, more studies have been published, and the body of evidence has grown, warranting a new evaluation. Furthermore, the methodology of the present study differed from the earlier study in several important respects. This analysis used different feasibility conditions, for example combining age groups, and applied quantitative meta-analysis methods to VE estimates in infants and children, with prespecified scenario analyzes and evaluation of populations who were partially vaccinated and vaccinated with at least one dose; it used a de-duplication strategy with only one study per country to avoid overlapping populations; and it integrated global RWE from immunization programs implemented with different vaccination schedules (for example, 2+1, 3+1) and program maturities. As such, this analysis offers an important addition to knowledge in this area.

The objective of this SLR was to evaluate the published RWE data on VE and VI of 4CMenB in infants, children and adolescents worldwide. The primary objective of this meta-analysis was to synthesize published RWE data to estimate the overall VE of 4CMenB against serogroup B-IMD in fully vaccinated infants and children, with secondary objectives of estimating VE in partially vaccinated individuals and those vaccinated with at least one dose (i.e., including both fully and partially vaccinated individuals).

## Methods

The SLR was conducted according to the Preferred Reporting Items for Systematic reviews and Meta-Analyses (PRISMA) standards^20^. The scope of the SLR was broader than the evaluation of VE and VI presented in the current study. For example, the SLR included multiple outcomes such as VE, VI, immunopersistence and immunogenicity, as summarized below (Table 1), not all of which were intended to be described in a single publication. The present SLR and meta-analysis intentionally focused only on the distinct but related topics of VE and VI. The full SLR scope is described for transparency.

**Table 1:** Selection criteria for inclusion in the systematic literature review

PICOS	Inclusion
Population	Infants, children/toddlers, adolescents, and young adults up to 25 years of age
Intervention(s)	MenB (including Bexsero, Trumenba) vaccines
Comparator(s)	Not restricted
Outcomes	Vaccine impact against IMD (e.g., incidence rates, rate ratios, odds ratios, hazard ratios or percent differences to compare outcomes before and after the implementation of a meningococcal vaccine program or campaign) Vaccine effectiveness against IMD (vaccine effectiveness defined e.g., as the prevalence of IMD during vaccinated periods/of vaccinated cohort to the prevalence of IMD during unvaccinated periods/of unvaccinated cohort) Immuno-persistence (e.g., antibody persistence, antibody response, immunity/immune response, duration/period of response/immunity) Immunogenicity (e.g., vaccine immunogenicity, antigenicity, serum bactericidal activity, human serum bactericidal activity (hSBA)) Duration of protection against IMD
Study design^a^	Real-world evidence (including case-control studies, prospective/retrospective cohort studies, ecological studies, negative case-control, screening analysis)
Timeframe	Manuscripts published in the last 10 years Conference abstracts published in year 2021 onwards
Other restrictions	Language – not restricted Geographical scope – not restricted

*hSBA* human serum bactericidal activity, *IMD* invasive meningococcal disease.

aCase reports, editorials, letters, reviews were excluded. Published SLRs and NMAs were flagged.

### Search strategy

Electronic database searches were conducted on November 8, 2024, in Medline and Embase (both accessed via the OVID platform), National Institutes of Health ClinicalTrials.gov (http://www.clinicaltrials.gov/), and conference proceedings from 2021 to 2024 indexed in Embase. Additional hand searches were conducted to identify conference proceedings not indexed in Embase and the most up-to-date SLRs. Search strategies used both controlled vocabulary (MeSH and EMTREE terms) and free-text terms related to all MenB vaccines. Full details of the Medline and Embase search strategies are provided in Supplementary Table 1.

### Selection criteria

Studies published in the last 10 years, and conference abstracts published in the last 3 years, were selected for inclusion based on Population, Intervention, Comparators, Outcomes, Study design (PICOS) criteria, summarized in Table 1. There were no restrictions by language or geography.

Titles and abstracts were screened for relevance by two independent reviewers, with differences resolved by consensus or a third reviewer if necessary. The full texts of articles classed as relevant by the screening process were then assessed by two independent reviewers, and again, differences were resolved by consensus or a third reviewer if necessary. References selected for inclusion were then cross-checked to see if different articles originated from the same study, and their citations searched to identify any missing studies.

As described above, the analysis presented here considered only studies reporting VE and VI. RWE studies on these topics (including case-control studies, prospective/retrospective cohort studies, ecological studies, negative case-control, screening analyzes) were included in this review. From this set of studies included in the review, a smaller set was then selected for inclusion in the meta-analysis, as described below.

### Data extraction

Data were extracted from the included studies by one reviewer and verified by a second reviewer. Extracted data included publication details, study details, baseline characteristics, and results for VE, VI, immunogenicity and immunopersistence (Supplementary Table 2). As stated above, the scope of the SLR was broader than the current study, which evaluated data on VE and VI only. In this analysis, VE was defined as the measure of direct protection against serogroup B-IMD conferred on vaccinated individuals, as reported in the original studies. VE estimates reflect the relative reduction in the risk of IMD among vaccinated compared to unvaccinated individuals, based on the effect measures provided by each study. VI describes the overall reduction in IMD incidence observed at the population level following the introduction of a meningococcal vaccination program, expressed as changes in disease incidence or related effect measures after program implementation.

After data extraction, the included studies were assessed using the Newcastle–Ottawa Scale for observational studies^21^. The tool evaluated studies on three domains: selection of study groups, comparability of groups, and ascertainment of exposure and outcomes. Two reviewers independently appraised each study, and discrepancies were resolved by consensus or by a third reviewer. All studies received high or moderate ratings and no studies were assessed as low quality. The quality assessment ratings for the studies selected for inclusion in the meta-analysis (see below), all of which were rated as high quality, are summarized in Supplementary Table 3.

### Meta-analysis

In this analysis, being fully vaccinated was defined as having received all 4CMenB vaccination doses for which the subjects were eligible based on their age and the local vaccination schedule, as reported in original studies. Partially vaccinated was then defined as not having completed the full 4CMenB vaccination schedule. Vaccinated with at least one dose was defined as having received at least one dose, and included both partially and fully vaccinated individuals.

For the meta-analysis, a feasibility assessment was conducted based on the studies included in the SLR to identify outcomes and populations with sufficient data for meta-analysis. As a result, meta-analysis for VE against serogroup B-IMD was feasible only in infants and children as a combined population. A meta-analysis in adolescents was not possible due to a lack of data, with only a single study reporting relevant estimates. Similarly, a meta-analysis of VI data could not be conducted because of major heterogeneity in the available studies in characteristics such as recruited population, vaccine eligibility, different pre- and post-vaccination durations, and differences in vaccination programs and coverage.

A total of six studies were selected for inclusion in the meta-analysis for VE against serogroup B-IMD in infants and children, of which five studies were included in the primary analysis, and one additional study was included in sensitivity analysis. These studies are summarized in Table 2. All studies employed retrospective designs, including screening and case-control studies, and one study estimating VE using incidence modeling. Study follow-up durations ranged from 2 to 7 years. Where multiple studies were available in a single country, only one study per country was selected for inclusion in the meta-analysis to avoid duplication, preferring studies with longer duration and/or wider coverage. Using these criteria, in the state of South Australia, Wang et al. 2023^22^ was selected for inclusion in the meta-analysis over the Wang et al. 2022 publication^23^. It should be noted that the Wang et al. 2025 study^24^ was published after this analysis had been completed; including it would have required repeating the search process, which was beyond the scope of the current analysis, and it was therefore not included. In England, the study selected was Argante et al. 2021^25^, because it had a longer duration than the Parikh et al. 2016 study^26^. It was selected over the Ladhani et al. 2020 study^27^ because it was a methodological reassessment of data from the Ladhani et al. 2020 study^27^ using an incidence modeling approach, which Argante et al. 2021^25^ showed to be more appropriate in settings with low disease incidence and high vaccine uptake than the screening method used in previous analyzes. The incidence modeling approach explicitly incorporated vaccinated and unvaccinated person-time data on population eligible and not eligible for vaccination, allowing adjustment for age, time and number of vaccine doses received, and provided more precise and statistically robust VE estimates that were consistent with previously observed reductions in disease incidence. Therefore, Argante et al. 2021^25^ was considered the most appropriate study on VE to include from the English surveillance data. In Italy, the study selected was Lodi et al. 2023^28^, because it had a longer duration and covered more Italian regions than the Azzari et al. 2020 study^29^. In Spain^30^, Portugal^31^, and Scotland^32^, the single study available in each country was included. The study in Scotland^32^ did not directly report VE data, so VE estimates were calculated as described below and used in scenario analysis.

**Table 2:** Studies selected for inclusion in the meta-analysis

Study	Country	Time period	Vaccination status			Multiple results selection	Vaccination program in country of study
			Full	Partial	≥1 dose		
Europe							
Argante 2021^25^	England	2011–2018	✓	✓		-	Program timelines: 2015 to 2018 Infant vaccination schedule: 2+1 schedule, doses at 8 week, 16 week, 12 month Catch-up program for 3 month and 4 month: 2+1 schedule at 3, 4, 12 month or 2 doses at 4, 12 month
Castilla 2023^30^	Spain	2015–2019	✓	✓	✓	VE against MenB estimated in main analysis (other serogroups and sensitivity analyzes were excluded)	Program timelines: 2015-10-05 to 2019-10-06 Infant vaccination schedule: 1st dose at 2 month 2nd dose ≥60 days later (or interval between doses ≥30 days if 3 primary doses at age 3-5 months) booster dose: if 1st dose was at <6 month: 12–15 month of life if 1st dose was at ≥6 month: 2nd year of life Vaccination start at ≥24 month of age: 2 doses with ≥30 days interval between doses
Lodi 2023^28^	Italy	2014– 2019	✓	✓	✓	VE estimated for 6 regions with case-control method (screening method based on 3 regions was excluded)	Italian vaccination programs (start date): Tuscany (2014): doses at 2, 3, 5 and 12 month Veneto (2015): doses at 6, 8, and 14 month Piedmont (2017): doses at 2, 4, 6, and 14 or 17 month since September 2018, doses at 3, 5, and 14 or 17 months since July 2020, doses at 2, 4, 12 or 14 month Liguria (2015): doses at 2, 3, 5, and 14 month since 2019, doses at 3, 5, and 14 month Sicily (2015): doses at 2, 3, 5, and 15 month Apulia (2014): doses at 2, 3, 5 and 14 month since 2021, doses at 2, 4, and 14 month
Rodrigues 2020^31^	Portugal	2014–2019	✓		✓	VE against MenB (other serogroups and sensitivity analysis were excluded)	Portuguese Society of Paediatrics recommendations: Infant vaccination schedule: First dose at 2 month Second dose by 4–5 month booster dose >12 month Vaccination start at >12 month: 2 doses at >12 month with no booster needed
Rodrigues 2023^32^	Scotland	2015–2022	VE of full and ≥1 dose vaccination were estimated based on reported data and included in sensitivity analysis			-	Program timelines: 2015 to 2022 Infant vaccination schedule: 2+1 schedule, doses at 8 week, 16 week, 12 month
Australia							
Wang 2023^22^	Australia	2018–2021	✓	✓		VE estimated with case-control method (screening method was excluded as providing less conservative results with higher point estimate and narrower confidence interval)	South Australia serogroup B meningococcal immunization program: Childhood programs (start October 2018) age ≤12 month: 2 primary doses + 1 booster (at 6 week, 4 month, 12 month, or interval between doses ≥2 month) (ongoing childhood program) age >12 month – <4 year: 2 doses (catch-up childhood program, end December 2019) Adolescent and young adult programs (start February 2019) age ~15–16 years: 2 doses (ongoing adolescent [year 10] school-based program) age ~16–17 years: 2 doses (catch-up adolescent [year 11] school-based program, end December 2019) age 17 to <21 years: 2 doses (catch-up young adult program, end February 2020)

*VE* vaccine effectiveness.

All studies reported either VE estimates or data enabling calculation of VE (Table 2). The VE used for input data in the meta-analysis was as reported in the included studies, with the numerical values and associated 95% confidence intervals (CI). All VE estimates were converted from percentages to log odds ratios (ORs), with standard errors derived from the CI, to enable pooling of effect sizes. The log ORs were then back-transformed to percentages and displayed in forest plots. No other data transformation or handling of missing data was applied. No assessment of the impact of missing data on the risk of bias was made, and no additional assessment of the body of evidence was made beyond the scope of the SLR.

For the study in Scotland^32^, VE estimates for input data into the meta-analysis were calculated using the screening method^33^, supplemented with data on vaccine coverage retrieved from Public Health Scotland reports^34^. Following this method, VE was estimated using the formula below, where PCV is the proportion of cases that are vaccinated and PPV is the proportion of population vaccinated (vaccine coverage)^33^:

1 $${VE}=1-\frac{\left({PCV}\,x\,\left(1-{PPV}\right)\right)}{\left(\left(1-{PCV}\right)\,x\,{PPV}\right)}$$

To include information from Rodrigues et al. 2023^32^, which reported the number of unvaccinated cases without distinction regarding vaccine eligibility (i.e., including cases among children too old for vaccination when the program started), an appropriate correction for the vaccine coverage and proportion of the unvaccinated population throughout the duration of the vaccination program was applied. Briefly, vaccine coverage in non-eligible cohorts was assumed to be 0, which was accounted for in the estimation of mean vaccine coverage and mean proportion of unvaccinated population across reported age groups. The detailed calculations are provided as an Excel file in Supplementary Data 1. These calculated VE estimates were used in scenario analysis.

The meta-analysis was conducted specifically in infants and children, as sufficient data were available only for this population. Five studies directly reported VE estimates (Table 2), and these data were pooled using random-effects and fixed-effect meta-analysis in the primary analysis. Frequentist methods were employed. Studies were weighted using the inverse-variance method. In random-effects meta-analyzes, the between-study variance was estimated using the DerSimonian-Laird method^35^. All analyzes were performed using R Statistical Software (v4.4.2)^36^. Random-effects models were considered a primary approach because all effects were estimated using non-randomized comparisons (thus, elevated heterogeneity was anticipated), and the results of the fixed-effect models were also presented for transparency. Heterogeneity was assessed using Cochran’s *Q* test and quantified with the *I*² statistic, with substantial heterogeneity defined as either a *Q* test *p*-value < 0.10 or *I*² > 50%, in line with established guidelines^37,38^. The primary analysis evaluated data from fully vaccinated populations.

Sensitivity analyzes (Supplementary Fig. 1) were conducted to test the robustness of the results as follows:

Scenario 1: Meta-analysis using the same approach as the primary analysis, but excluding Rodrigues et al. 2020 in Portugal^31^ (which enrolled a broad population from infancy to adolescence, with unvaccinated cases potentially including adolescents) and Argante et al. 2021 in England^25^ (which included vaccinated cases aged 2 months–2 years and unvaccinated cases from 0 months to ≥45 years);

Scenario 2: Meta-analysis as in the primary analysis, but including an additional VE estimate for Scotland. Rodrigues et al. 2023^32^ in Scotland did not report VE directly, but allowed estimation using the screening method for ages 0≤5 years at IMD onset;

Scenario 3: Subgroup meta-analysis comparing studies that included both vaccine-eligible and ineligible cohorts, Argante et al. 2021 in England^25^ and Rodrigues et al. 2023 in Scotland^32^ versus those with only vaccine-eligible cohorts;

Scenario 4: Meta-analysis restricted to studies from countries with the same 2+1 vaccination schedule (Argante et al. 2021^25^ in England and Rodrigues et al. 2023^32^ in Scotland) to evaluate consistency of VE in similar immunization settings.

Secondary analyzes evaluated VE for partially vaccinated populations and those vaccinated with at least one dose (Supplementary Fig. 1).

The study has been registered (https://www.gsk-studyregister.com/trials/224096). No protocol amendments were made.

## Results

### Systematic literature review

**Fig. 1:**
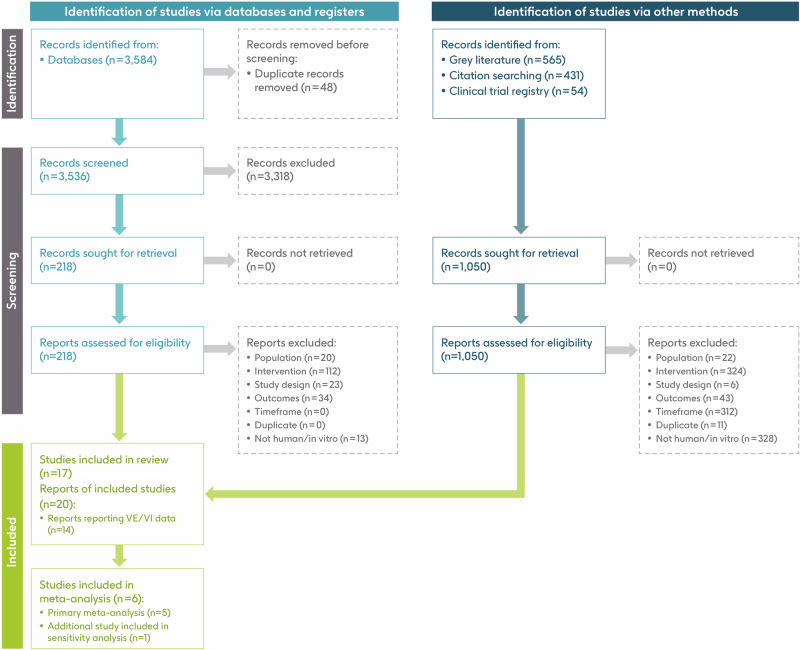
PRISMA flow diagram. PRISMA Preferred Reporting Items for Systematic reviews and Meta-Analyses, VE vaccine effectiveness, VI vaccine impact.

A total of 4575 records remained after removal of duplicates and were screened based on their titles and abstracts. Full texts of 1268 publications were reviewed, and after removal of one reference that did not contain any new data, 17 studies reported across 20 publications were included in the SLR. Of these 20 publications, 14 reported data on VE and/or VI^22,23,25–31,39–43^ and are included here (Fig. 1).

### Vaccine effectiveness

Thirteen publications^22,23,25–31,39–42^ reported data on 4CMenB VE. Of these, ten were considered primary studies: one in Australia^22^; two in Italy^28,29^; one in Spain^30^; four in the UK^25–27,39^; one in Portugal^31^; and one in Canada^40^. In addition, a study in Scotland did not directly report VE data but did report data from which VE estimates could be calculated for use in a scenario analysis in the meta-analysis^32^. One study identified through the SLR reported only data on VE against serogroup W^39^, and was therefore outside of the scope of the meta-analysis (which considered serogroup B-IMD only). The other nine studies (12 references) reported data on VE of 4CMenB against serogroup B-IMD (Table 3). Of these nine studies, one was conducted in infants only, five in infants and children, and three in infants, children and adolescents (Table 3).

**Table 3:** Studies reporting data on vaccine effectiveness of 4CMenB against serogroup B-IMD

Study name	Study type/study period/vaccine	Population	Vaccination status	VE, % (95% CI)
Australia				
Wang 2023^22^ * Australia	• Cohort • 2012–2021 • 4CMenB	Infants and children	1 dose	47.9 (0–91.3)
			2 doses	93.2 (29.3–99.3)
		Adolescents	2 doses	83.5 (0–98.2)
	• Case-control • 2012–2021 • 4CMenB	Infants and children	1 dose	40.6 (0–90.4)
			2 doses	90.7 (6.9–99.1)
		Adolescents	2 doses	89.4 (0–99.0)
		Adolescents (>10–25 years)	2 doses	100 (NR)^^
	• Cohort, Case-control • 2011–2019 • 4CMenB	Children (3 years)	2+1 (3-doses)	100 (NR)^^
Wang 2022^23^* Australia	• Cohort • 2011–2019 • 4CMenB	Vaccine-eligible infants and children	2 doses	94.2 (36.6–99.5)
	• Case-control • 2011–2019 • 4CMenB	Vaccine-eligible infants and children	2 doses	94.7 (40.3–99.5)
	• Cohort, Case-control • 2011–2019 • 4CMenB	Children (>1–10 years)	2+1 (3-doses)	100 (NR)^^
		Adolescents (>10–25 years)	2 doses	100 (NR)^^
Europe				
Argante 2021^25^** United Kingdom	• Incidence modeling based on cohort • 2011–2018 • 4CMenB	Infants in UK^’^	1 dose^’^	33.5 (12.4–49.7)
		Infants, children in UK^’^	2 doses^’^	78.7 (71.5–84.5)
			3 doses^’^	80.1 (70.3–86.7)
Azzari 2020^29^ Italy	• Cohort • 2014–2018 • 4CMenB	Infants and children 0–5 years of age (Tuscany)	Overall	93.6 (55.4–99.1)
		Infants 0–1 year of age (Tuscany)	Overall	82.2 (−70.3–98.1)
	• Cohort • 2015–2018 • 4CMenB	Infants and children 0–4 years of age (Veneto)	Overall	91 (59.9–97.9)
		Children 1–2 years of age (Veneto)	Overall	50.9 (−274.4–93.5)
Castilla 2023^30^ Spain	• Case-control • 2015–2019 (VE in preventing IMD caused by serogroup B) • 4CMenB	Infant and children <5years of age, born/residing in Spain	≥1 dose	64 (41–78) 64 (39–78)^a^
			Partially vaccinated	50 (3–75) 47 (−5–73)^a^
			Fully vaccinated	71 (45–85) 73 (47–86)^a^
Ladhani 2020^27^** United Kingdom	• Cohort • 2015–2018 • 4CMenB	Infants and children <5 years of age	1 dose	24.1 (−37.6–58.2)
		Infants and children <5 years of age	2 doses	52.7 (−33.5–83.2)
			3 doses	59.1 (−31.1–87.2)
		Infants and children <5 years of age with MATS positive strain	2 doses	64.4 (NR)
			3 doses	71.2 (NR)
Lodi 2023^28^ Italy	• Case-control • 2006–2020 • 4CMenB	Infants and children <6 years of age (Apulia, Liguria, Piedmont, Sicily, Veneto, Tuscany)	Overall	87.9 (53.7–96.4)
			Partially vaccinated	91.4 (36.0–99.3)
			Only 1 dose	100 (60.6–100)
			≥1 dose	92.4 (67.6–97.9)
			Fully vaccinated	95.6 (71.7–99.1)
	• Cohort • 2006–2020 • 4CMenB	Infants and children <6 years of age, additional analysis (Tuscany, Veneto, Piedmont)	Overall	75.0 (0.2–93.3)
			≥1 dose	85.7 (25.9–96.7)
			Fully vaccinated	91.7 (24.4–98.6)
		Infants and children <6 years of age (Tuscany, Veneto, Piedmont)	Fully vaccinated	94.9 (83.1–98.4)
		Infants and children <6 years of age (Tuscany)	Fully vaccinated	93.6 (55.4–99.1)
		Infants and children <6 years of age (Veneto)	Fully vaccinated	93.5 (71.7–98.5)
		Infants and children <6 years of age (Piedmont)	Fully vaccinated	100 (70.7–100)
Parikh 2016^26^** United Kingdom	• Cohort • 2015–2016 • 4CMenB	Infants 2–4 months of age’	2 doses’	82.9 (24.1–95.2)
			≥1 dose’	64 (8.9–84)
		Infants 2–4 months of age’	1 dose’	22 (−105–67.1)
Rodrigues 2020^31^ Portugal	• Case-control • October, 2014–March, 2019 • 4CMenB	Infants, children and adolescents with ≥134 days of age	Fully vaccinated	79^’^ (OR: 0.21 (0.08–0.55)
		Infants, children and adolescents with ≥74 days of age	≥1 dose	82^’^ (OR: 0.18 (0.08–0.44)
North America				
De Wals 2017^40^ Canada	• Cohort • 2006–2016^ • 4CMenB	Infants, children, adolescents Residents in SLSJ region (Quebec)	Vaccinated	100 (NR)^#^
Deceuninck 2019^41^ Canada	• Cohort • 2006–2018 • 4CMenB	Infants, children, adolescents: Residents in SLSJ region (Quebec)	Vaccinated	79 (−231–99)
Martinón-Torres 2021^42^ ***^ Canada	• Cohort • 2006–2016# • 4CMenB	Infants, children, adolescents Residents in SLSJ region (Quebec)	Vaccinated	100 (NR)^#^
	• Cohort • 2006–2019^ • 4CMen	Infants, children, adolescents: Residents in SLSJ region (Quebec)	Vaccinated	59 (−352– 96)^

Although the Rodrigues 2023^32^ study do not report on VE, reported data enabled VE calculation. The study was included in the sensitivity analysis.

*CI* confidence interval, *IMD* invasive meningococcal disease, *MATS* Meningococcal Antigen Typing System, *SLSJ* Saguenay-Lac-Saint-Jean, *VE* vaccine effectiveness.

*Linked references.

**Linked references.

***Linked references.

^ Data for 5-year post vaccination.

# Data for 2-year post vaccination.

‘Assumed for^31^ based on OR.

^^Based on zero cases reported in adolescents receiving 2-dose and children receiving 2+1 schedule (>1 to 25 years).

“The population is assumed based on the age at which the doses were given, but it is not clearly stated in the publication.

^a^The sensitivity analysis was limited to children 134 to 1825 days of age and those who were unvaccinated against serogroup C meningococcus or had high-risk conditions.

For a full vaccination schedule in infants and children (defined as having received all 4CMenB vaccination doses for which the subjects were eligible based on their age and the local vaccination schedule, as reported in original studies), VE estimates ranged from 59.1% (95% CI: −31.1, 87.2) to 100% (95% CI not reported) (Table 3). Only one study, published in two references^22,23^, reported VE data specifically in adolescents. For two doses, the VE ranged from 83.5% (95% CI: 0, 98.2) to 100% (95% CI not reported) (Table 3). Two other studies reported data across combined populations, including adolescents^31,40^ (Table 3).

### Vaccine impact

Eleven references, representing eight primary studies, reported data on VI against serogroup B-IMD and are summarized in Supplementary Table 4.

In infants aged 12 weeks to 11 months in the state of South Australia, VI was 63.1% (adjusted incidence rate ratio [IRR] 0.369, 95% CI: 0.191 to 0.710) over a 3-year observation period, and 60% (adjusted IRR 0.4, 95% CI: 0.23, 0.69) over a 2-year period. In adolescents aged 15–18 years in the state of South Australia, the VI was 78.5% (adjusted IRR 0.215, 95% CI: 0.069, 0.670) over a 3-year period, and 73% (adjusted IRR 0.27, 95% CI: 0.06, 1.16) over a 2-year period.

A study in the UK conducted soon after implementation of a vaccination program in infants using a 2+1 schedule with doses at 2, 4 and 12 months of age^26^ reported that the VI was 47% (IRR 0.53, 95% CI: 0.33, 0.87) in infants aged ≥18 weeks vaccinated with two doses. A further study in the UK reported that adjusted IRR remained unchanged for 2017–2018 compared with 2010–2015 in infants aged 9–17 weeks who were too young to be eligible for vaccination (0.99, 95% CI: 0.56, 1.74), whereas in the vaccine-eligible cohort, infants aged 18–51 weeks, the VI was 70% (adjusted IRR 0.3, 95% CI: 0.19, 0.49)^27^. In Italy, VI in vaccinated infants and children reached 93–94% in Tuscany, 84–90% in Veneto, and 100% in Piedmont, and pooled analysis indicated a 90% (95% CI: 75% to 97%) VI in vaccinated individuals under 6 years of age.

In Canada^40^, the VI for all ages (infants, children, adolescents and young adults) combined was 67% in Quebec, 92% in Saguenay-Lac-St-Jean and 53% in other regions (IRR 0.33 in Quebec, 0.08 in Saguenay-Lac-St-Jean and 0.47 in other regions, 95% CI not reported).

### Meta-analysis

**Fig. 2:**
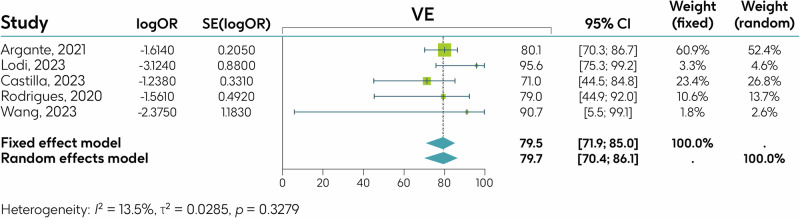
Vaccine effectiveness of 4CMenB against serogroup B-IMD in fully vaccinated infants and children (primary analysis). Error bars indicate 95% CI. CI confidence interval, IMD invasive meningococcal disease, OR odds ratio, SE standard error, VE vaccine effectiveness.

The primary meta-analysis was based on data from five studies^22,25,28,30,31^. VE reported by the individual studies ranged from 71% (95% CI: 45, 85)^30^ to 95.6% (95% CI: 71.7, 99.1)^28^ (number of decimal places as reported in original publications). The meta-analysis estimated the pooled VE in fully vaccinated infants and children at 79.7% (95% CI: 70.4, 86.1) using the random-effects model and 79.5% (95% CI: 71.9, 85.0 using the fixed-effect model (Fig. 2). According to the pre-specified criteria, there was no substantial heterogeneity between the studies (*I²* = 13.5%, *p* = 0.3279) (Fig. 2).

**Fig. 3:**
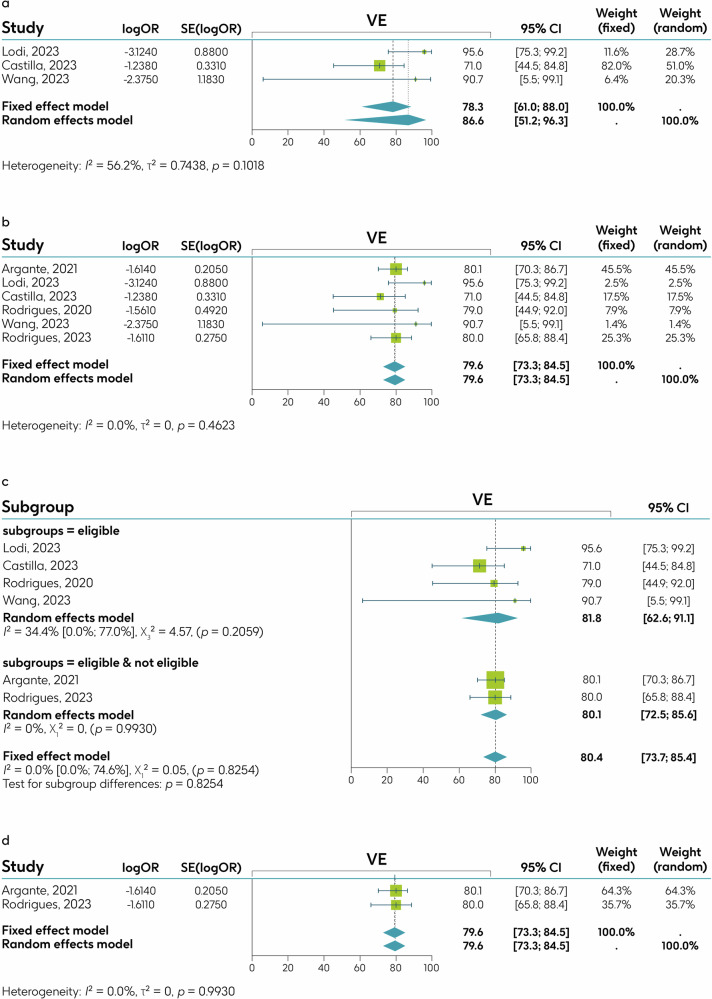
VE of 4CMenB against serogroup B-IMD in fully vaccinated infants and children, sensitivity analyzes. **a** meta-analysis excluding two studies in which unvaccinated comparator groups included adolescents or adults^25,31^ (Scenario 1); **b** meta-analysis including an additional VE estimate calculated based on data from a study in Scotland^32^ (Scenario 2); (**c**) subgroup meta-analysis comparing studies including only vaccine-eligible cohorts versus subgroup of studies including mixed population of vaccine-eligible and vaccine-ineligible cohorts (Scenario 3); **d** meta-analysis limited to studies from countries with the same 2+1 vaccination schedule (Argante et al. 2021^25^ in England and Rodrigues et al. 2023^32^ in Scotland (Scenario 4). Error bars indicate 95% CI. CI confidence interval, IMD invasive meningococcal disease; OR odds ratio, SE standard error, VE vaccine effectiveness.

The first sensitivity analysis (Scenario 1) excluded two studies in which unvaccinated comparator groups included adolescents or adults^25,31^ (Fig. 3a). This analysis resulted in a pooled VE under the random-effects model of 86.6% (95% CI: 51.2, 96.3) and 78.3% (95% CI: 61.0, 88.0) using the fixed-effect model. In this scenario, moderate heterogeneity was observed (*I²* = 56.2%, *p* = 0.1018), supporting the use of the random-effects model. It is also noteworthy that this scenario excluded the largest contributing study in the primary analysis^25^, and exclusion of this study did not materially alter the VE estimates, which remained consistent with the primary analysis.

A second sensitivity analysis added calculated VE data from an additional study in Scotland^32^ to expand the evidence base and broaden the geographic scope (Scenario 2) (Fig. 3b). This analysis produced a pooled VE from both random-effects and fixed-effect models of 79.6% (95% CI: 73.3, 84.5). No between-studies heterogeneity was detected (*I²* = 0%, *p* = 0.4623).

A subgroup analysis (Scenario 3) grouped studies based on whether they included only vaccine‑eligible cohorts (4 studies^22,28,30,31^), or a broader population that also included vaccine-ineligible individuals (2 studies^25,32^), including cases from the pre‑vaccination period and/or children too old for vaccination when the program began (Fig. 3c). There was no statistically significant difference between the pooled VE estimates for the subgroups: 81.8% (95% CI: 62.6, 91.1) for vaccine-eligible cohorts and 80.1% (95% CI: 72.5, 85.6) for mixed populations (*p* = 0.8254). No substantial between-studies heterogeneity was observed within either subgroup (vaccine-eligible-only subgroup *I²* = 34.4%, *p* = 0.2059; mixed population subgroup *I²* = 0%, *p* = 0.9930).

A further sensitivity analysis (Scenario 4) used only data from the studies conducted in England and Scotland^25,32^, as both countries had implemented the same 2+1 schedule with doses at 2, 4 and 12 months of age (Fig. 3d). This analysis produced a pooled VE estimate from both fixed-effect and random-effects models of 80.1% (95% CI: 72.5; 85.6). No between-studies heterogeneity was detected (*I²* = 0%, *p* = 0.9930).

These sensitivity analyzes further supported the robustness of the primary analysis results, showing minimal variation despite differences across studies in the inclusion of vaccine-ineligible cohorts (Scenario 3) and broader unvaccinated populations extending into adolescence and adulthood (Scenario 1), after supplementing the analysis with estimated VE data for another country (Scotland, Scenario 2), or after restricting the analysis to countries with the same 2+1 vaccination schedule (Scenario 4).

**Fig. 4:**
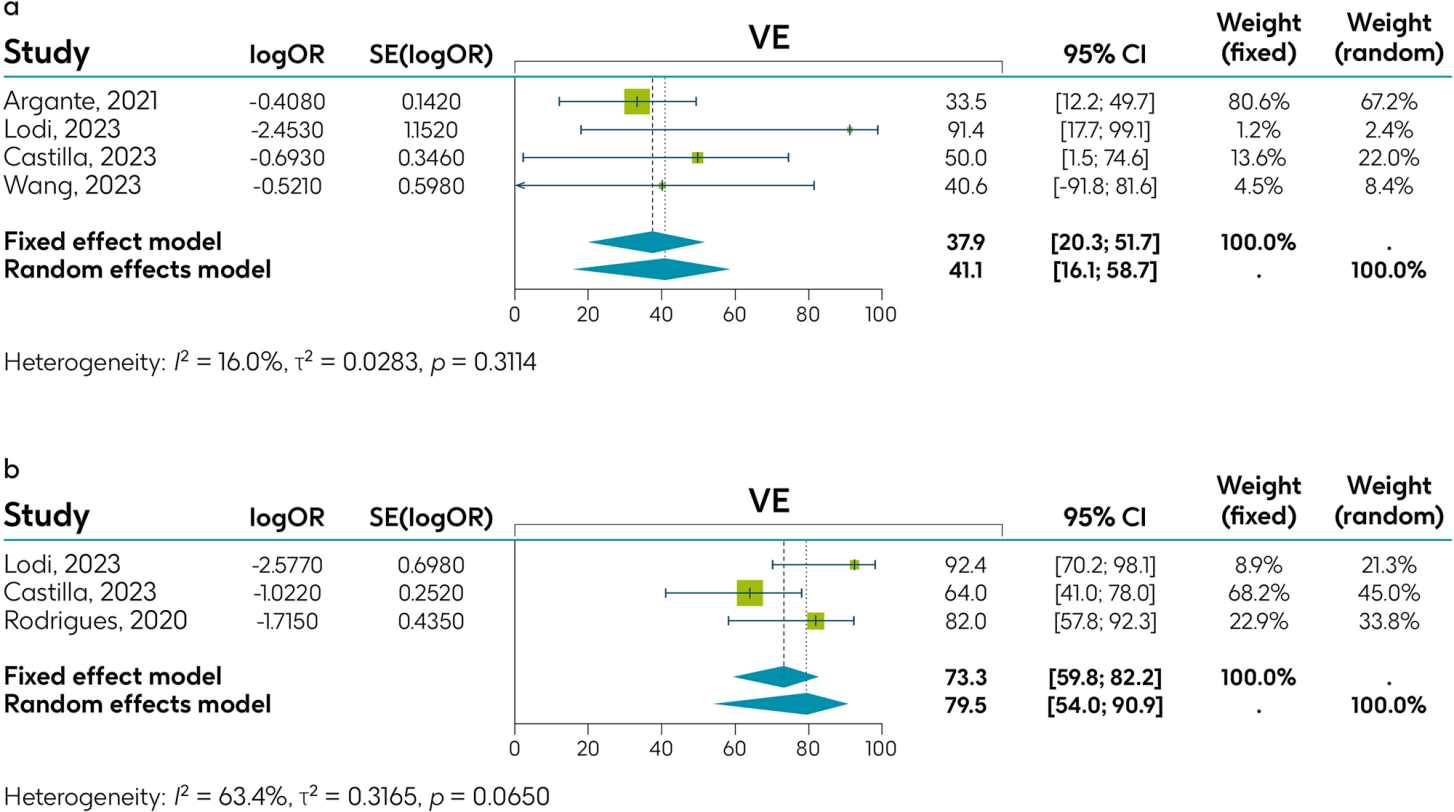
VE of 4CMenB against serogroup B-IMD in infants and children, secondary analyzes. **a** in partially vaccinated infants and children; **b** in infants and children who had received at least one dose. Error bars indicate 95% CI. CI confidence interval, IMD invasive meningococcal disease, OR odds ratio, SE standard error, VE vaccine effectiveness.

The meta-analysis in partially vaccinated infants and children (defined as not having completed the full 4CMenB vaccination schedule as reported in the original studies) (Scenario 5) was based on data from four eligible studies and estimated the pooled VE under the random-effects model at 41.1% (95% CI: 16.1, 58.7) and 37.9% (95% CI: 20.3, 51.7) under the fixed-effect model (Fig. 4a). There was no substantial heterogeneity between the studies (*I²* = 16.0%, *p* = 0.3114) (Fig. 4a). This result indicated that VE in partially vaccinated individuals appeared to be substantially lower than reported in the primary analysis for fully vaccinated individuals.

The analysis in infants and children who had received at least one dose of 4CMenB (i.e., including partially and fully vaccinated individuals) (Scenario 6) was based on data from three studies. The pooled VE with the random-effects model was 79.5% (95% CI: 54.0, 90.9) and 73.3% (95% CI: 58.8, 82.2) with the fixed-effect model (Fig. 4b). Heterogeneity was moderate (*I²* = 63.4%, *p* = 0.0650), supporting the use of the random-effects model. This VE estimate was similar to the VE estimated in the primary analysis, indicating that at least one dose of the vaccine provides a substantial level of protection, although the CI was wider than in the primary analysis, indicating a higher level of uncertainty in the result. The similarity between the estimates from Scenario 6 and the primary analysis reflected the dominance of data from fully vaccinated individuals, who represented the largest group in the data included in the meta-analysis.

Adding the calculated VE data from the study in Scotland to the analysis in infants and children who had received at least one dose of 4CMenB (Scenario 7) resulted in a VE estimate of 73.5% (95% CI: 56.8, 83.8) using the random-effects model and 69.7% (95% CI: 59.0, 77.6) using the fixed-effect model. Heterogeneity was moderate (*I²* = 52.1%, *p* = 0.099), supporting the use of the random-effects model. The VE estimate from the random-effects model was numerically lower than that derived from Scenario 6, but had a narrower CI, indicating greater precision.

## Discussion

To our knowledge, this is the first meta-analysis to be conducted of data on VE against serogroup B-IMD for the 4CMenB vaccine. Reported VE data from five studies were included in the primary analysis, with calculated VE estimates from a sixth study added in scenario analysis. The primary analysis estimated the pooled VE in fully vaccinated infants and children at 79.7% (95% CI: 70.4, 86.1) using the random-effects model.

Vaccine impact was high in the identified studies. For example, in Italy, the relative case reduction in vaccinated infants and children aged <6 years was reported at 93% in Tuscany, 84% in Veneto, 100% in Piedmont and 90% across all three regions^28^. In the UK, the reduction in IMD cases was 60% in the overall population of infants aged <1 year^43^, and the reduction in IMD incidence was 75% in vaccine-eligible infants and children aged <5 years^27^. A meta-analysis of VI was not conducted as vaccine coverage is an important parameter affecting VI, in contrast to VE, and this introduced additional heterogeneity between studies. Vaccine coverage differed across the settings of the studies included in this review. For example, coverage in England was estimated at 92.5% for the primary two-dose immunization schedule and 87.9% for the third dose^25^, while in Italy coverage at age 24 months ranged from 81.4% in Piedmont to 83.9% in Tuscany^28^ and in Portugal two-dose coverage by the first birthday was 56.7% in the 2018 birth cohort^31^.

The primary meta-analysis included VE data reported by five studies in five different high-income countries (the state of South Australia, England, Italy, Spain and Portugal). VE estimates in the individual studies ranged from 71% (95% CI: 45, 85) in Spain^30^ to 95.6% (95% CI: 71.7, 99.1) in Italy^28^. This supports the high effectiveness of 4CMenB across high-income countries in a variety of geographical areas, and demonstrates the broad effectiveness of 4CMenB in reducing the disease burden of serogroup B-IMD across various countries and regions globally.

Sensitivity analyzes showed minimal variation in estimated VE for a range of scenarios, supporting the robustness of the VE estimates from the primary analysis and the generalizability of the results, and confirming the high effectiveness of 4CMenB in infants and children. The findings of the sensitivity analyzes indicate that 4CMenB has consistently high VE across high-income countries in different geographic regions (studies from Europe and Australia, with results unaffected by adding a further study in Scotland), different age ranges (studies included vaccination of infants and children), and different vaccination schedules (with results unaffected by limiting the analysis only to countries using the same 2+1 schedule). Furthermore, the scenario analysis (Scenario 1) that excluded data from the largest study in the primary analysis (the study from England^25^) reported VE estimates that were consistent with the primary analysis in both direction and magnitude. This indicates that the results were not driven by a single study, supporting the robustness of the analysis. The synthesis of the body of available RWE on VE for 4CMenB against serogroup B-IMD in fully vaccinated infants and children presented in this meta-analysis can help to inform decision-making on vaccination recommendations.

Secondary analyzes indicated that subjects vaccinated with at least one dose showed VE similar to that estimated in the primary analysis. This finding should be interpreted with caution, as the analysis included individuals who were fully vaccinated for their age, and fully vaccinated individuals represented the largest group in the dataset. In a further secondary analysis in partially vaccinated individuals, VE appeared to be substantially lower than estimated in the primary analysis for fully vaccinated individuals. This finding of substantially higher VE in fully vaccinated infants and children than in partially vaccinated individuals supports the importance of aiming for completion of the full vaccination schedule in a timely manner to provide full protection from an early age. It should be noted that in the present study, “fully vaccinated” was defined based on the local vaccination schedule, as reported in the original publications. Vaccination programs may differ in the number and timing of vaccination doses, and therefore, the same number of doses could be considered as “fully vaccinated” in some programs and as “partially vaccinated” in other programs.

A previously reported SLR of real-world studies on 4CMenB VE and impact identified five studies, four of which reported VE data, but found that quantitative analysis could not be applied due to heterogeneity in the studies, reflecting variations in vaccine strategies in the study settings^19^. The present analysis conducted a new and independent SLR and identified a larger number of studies, several of which had been published since the earlier SLR was conducted. By including only one study per country, to avoid duplication, we identified a group of five studies reporting VE data in infants and children that were sufficiently similar to be included in a meta-analysis, plus an additional study from which VE data could be calculated for inclusion in a scenario analysis. Of the six studies included in the present meta-analysis, only one^31^ was also included in the earlier SLR^19^. All four of the VE studies in the earlier SLR were also identified in the present SLR, but three of the four were not included in the present meta-analysis, two because alternative studies from the same countries were available for use instead^27,29^ and one because its VE estimate was not based on cases vaccinated in infancy^41^. A recent meta-analysis^44^ led by the Standing Vaccination Committee at the Robert Koch Institute in Germany (STIKO), the German National Immunization Technical Advisory Group (NITAG)^45^, also showed comparable results for 4CMenB VE in children. This meta-analysis was part of STIKO’s evaluation and recommendation of 4CMenB for use in infants. In contrast to the earlier SLR^19^, which concluded that quantitative synthesis was not feasible, and to the STIKO meta-analysis, which focused on national decision-making in a single country, the present analysis integrated the most recent global RWE, applied de-duplication by country, and explored both fully and partially vaccinated infants and children across diverse program contexts.

Several of the studies included in this analysis reported VE over multiple years following the implementation of vaccine programs, consistently showing high and stable effectiveness estimates. For example, studies from the state of South Australia reported two-dose VE in infants using the case-control method of 94.7% after 2 years of program implementation and 90.7% after 3 years of implementation^22^, and recently published results after 5 years of implementation reported VE using the case-control method at 81.9% for two-dose vaccination in infants and children eligible for the two-dose program, and 98.8% for three-dose vaccination in infants and children eligible for the three-dose program^24^. These findings suggest sustained high effectiveness over time, although they do not specifically account for the time elapsed since vaccination. It should be noted that the present analysis included data from the 3-year South Australia study^22^, but not from the 5-year study^24^, because the 5-year study was not published until after the current analysis had been completed. A study conducted in Spain considered time elapsed since the last vaccine dose and reported no evidence of waning immunity, but this analysis was conducted on IMD caused by any serogroup (i.e., it was not specific to serogroup B-IMD), and distinguished only between <12 months elapsed and ≥12 months elapsed^30^. There is a need for a better understanding of the duration of protection offered by the 4CMenB vaccine. Data from ongoing surveillance programs monitoring IMD incidence and strain profile over time, particularly in high-risk populations such as infants and young children, would be valuable to help explore this issue.

This study has a number of strengths. The SLR conducted comprehensive searches across multiple databases, including conference abstracts for the last 3 years, without geographical or language restrictions. The SLR was also conducted in accordance with PRISMA guidelines. A methodologically rigorous meta-analysis was conducted following a comprehensive feasibility assessment, and sufficient studies were identified for meta-analysis to be feasible in infants and children for VE of 4CMenB against serogroup B-IMD. Extensive scenario analyzes were included to test the robustness of the results to a range of conditions, such as inclusion/exclusion of different studies, different vaccination schedules or the addition of calculated VE data. The studies included in the meta-analysis covered five different high-income countries, supporting widespread applicability for the results.

The study also has limitations. All the studies included in the review were retrospective observational studies. Given the rarity of IMD, prospective or randomized effectiveness studies are not feasible, and therefore, real-world observational studies represent the most appropriate evidence in this context. The retrospective studies included in this analysis were all population-based, supporting the interpretation of their findings despite the study designs. As with all real-world observational studies, the VE estimates included in this meta-analysis are subject to potential residual confounding and bias. Confounding by indication and healthy vaccinee bias may arise if vaccinated and unvaccinated children differ systematically in underlying risk factors that are incompletely measured or unmeasured in the source data. Misclassification of vaccination status, particularly in settings with fragmented immunization records, could also bias VE estimates towards or away from the null. In addition, changes over time in awareness of IMD, health-care seeking, diagnostic testing and reporting practices can influence observed incidence and vaccine impact. Although several of the included studies attempted to adjust for key confounders, and our scenario analyzes restricted to more homogeneous populations, residual bias cannot be fully excluded. A further limitation is that it was not possible for this analysis to assess VE by time since vaccination, as none of the studies identified captured individual-level data on VE by time since vaccination. IMD is uncommon, and the published RWE on 4CMenB has reported VE over time since the implementation of national vaccination programs, rather than time since vaccination for individuals. Most VE and impact data included in this review come from high-income countries with established surveillance systems and high infant vaccine coverage. There was a lack of identified data from low- and middle-income countries, where IMD epidemiology, access to care, and competing serogroups differ, and extrapolation to such settings should therefore be made with caution. Additional RWE from such settings would be valuable to inform global policy. Strengthening vaccination research in low- and middle-income settings across a range of vaccines and diseases would help to expand the global evidence base for vaccination. In addition, the studies identified in the SLR displayed considerable heterogeneity, with differences in factors such as vaccine eligibility, study duration, vaccination programs, and vaccination coverage. This limited the feasibility of quantitative analysis, and only five studies were eligible for inclusion in the primary meta-analysis (plus a further study included in sensitivity analysis). Within these studies, there was still heterogeneity in factors such as the definitions of age groups, vaccination schedules and geographical region. While this variation strengthened the evidence base by including information from diverse contexts, it also introduced methodological heterogeneity that was addressed by conducting a range of scenario analyzes. As well as differences in vaccine schedules, many of the included studies reported combined data across infants and young children, and thus it was not feasible in this analysis to derive separate pooled VE estimates for specific vaccination schedules, with the exception of the analysis of 2+1 vaccination in England and Scotland reported in Scenario 4. Meta-analysis was feasible only for one age group (infants and children) and was not possible in adolescents because only one study was available with adolescent-specific VE data. Only VE against serogroup B-IMD could be analyzed, due to a lack of available data on other serogroups. The studies included in the meta-analysis were conducted in diverse high-income countries with differing MenB strain distributions. Because VE in each study reflects protection against the mix of circulating strains, some of which may not be vaccine-preventable, comparisons across settings and pooled estimates should be interpreted with caution. In two of the studies^22,32^, the study period covered part of the COVID-19 pandemic period, which could have reduced the incidence of IMD, although, as the pandemic period comprised a relatively short part of the study periods, this is unlikely to have had an important impact on the results.

This meta-analysis provides quantitative evidence of 4CMenB VE of approximately 80% against serogroup B-IMD in fully vaccinated infants and children across a range of geographical locations and various schedules. Similar results were obtained across a range of scenarios, supporting the robustness and generalizability of the VE meta-analysis results in high-income countries or similar settings. In adolescents, vaccine impact and effectiveness evidence was summarized qualitatively due to heterogeneity and limited age-specific data. A study conducted in the state of South Australia reported high impact and effectiveness in adolescents. These findings support the potential role of 4CMenB vaccination in reducing the disease burden of serogroup B-IMD and provide reliable data to inform policy-making decisions.

## Supplementary information

Supplementary information

supplementary file: video script

Supplementary Data 1

Supplementary Data 1

## Data Availability

The datasets generated and/or analyzed during the current study are not publicly available due to confidentiality reasons but are available from the corresponding author on reasonable request.
