# Supplementary material for: Real-world effectiveness and impact of the 4CMenB vaccine against serogroup B invasive meningococcal disease: a systematic review and meta-analysis
Source: NPJ Vaccines. 2026 Jul 6;11:153. doi: 10.1038/s41541-026-01511-y (PMC13401604; doi:10.1038/s41541-026-01511-y)
Supplement: Supplementary file 1 — Supplementary information [file 41541_2026_1511_MOESM1_ESM.docx]

**Supplementary Material**

**Real-world effectiveness and impact of the 4CMenB vaccine against serogroup B invasive meningococcal disease: a systematic review and meta-analysis**

Pavo Marijic^1^, Katarzyna Jamróz-Dolińska^2^, Wojciech Margas^2^, Lucian Gaianu^3^, Gaurav Mathur^4^, Piotr Wojciechowski^2^, Thatiana Pinto^5^, Tia Vincent^3^, Helen Petousis-Harris^6^, Terry Nolan^7^, Federico Martinon-Torres^8-11^, Lee H Harrison^12^, Zeki Kocaata^1^

**Affiliations:**

^1^GSK, Munich, Germany

^2^Clever-Access, Kraków, Poland

^3^GSK, London, United Kingdom

^4^GSK, Rockville, Maryland, United States of America

^5^GSK, Wavre, Belgium

^6^University of Auckland, Auckland, New Zealand

^7^University of Melbourne, Melbourne, Australia

^8^Translational Pediatrics and Infectious Diseases, Hospital Clínico Universitario de Santiago (SERGAS) and University of Santiago de Compostela (USC), Santiago de Compostela, Galicia, Spain.

^9^Genetics, Vaccines and Pediatric Infectious Diseases Research Group (GENVIP), Instituto de Investigación Sanitaria de Santiago de Compostela (IDIS), Santiago de Compostela, Galicia, Spain.

^10^Centro de Investigación Biomédica en Red de Enfermedades Respiratorias (CIBERES), Instituto de Salud Carlos III, Madrid, Spain.

^11^WHO Collaborating Centre for Vaccine Safety, Santiago de Compostela, Galicia, Spain.

^12^Center for Genomic Epidemiology, University of Pittsburgh School of Medicine, Pittsburgh, Pennsylvania, United States of America

## Supplementary Table 1. Search strategy

**Search strategy for real-world evidence (RWE) run in Medline and Embase databases (via the OVID interface) on November 8, 2024**

| # | PICOS | Searches | Results |
| --- | --- | --- | --- |
| 1 | Intervention (I) | meningococcus vaccine?/ or meningococcal vaccine?/ or ((meningococcus or meningococcal) adj2 vaccin*).mp. | 14,967 |
| 2 |  | meningococcus/ and vaccin*.mp. | 8,834 |
| 3 |  | ((invasive meningococcal disease? or IMD or IMDs) adj2 vaccin*).mp. | 136 |
| 4 |  | (neisseria meningitidis/ or neisseria meningitidis.mp.) and vaccin*.mp. | 11,853 |
| 5 |  | meningococcus vaccine?/ or meningococcal vaccine?/ or ((meningococc* or meningitid*) adj3 vaccin*).mp. | 15,919 |
| 6 |  | exp vaccine?/ and (meningococc* or meningitid*).mp. | 18,063 |
| 7 |  | MenB-FHbp vaccine/ or menB/ or (menB or 4CMenB or MenB4C or meningococcal group-B vaccine? or (rMenB adj2 OMV) or trumenba or bexsero or rLP2086).mp. | 8,152 |
| 8 |  | (4CMenB or 4C MenB or MenB-4C).mp. | 996 |
| 9 |  | or/1-8 | 22,219 |
| 10 | Study design (S) | Epidemiologic studies/ | 293,885 |
| 11 |  | exp case control studies/ | 1,795,340 |
| 12 |  | exp cohort studies/ | 3,910,942 |
| 13 |  | Cross-sectional studies/ | 1,067,963 |
| 14 |  | Clinical study/ | 174,356 |
| 15 |  | Case control study/ | 566,602 |
| 16 |  | Family study/ | 25,865 |
| 17 |  | Longitudinal study/ | 401,890 |
| 18 |  | Retrospective study/ | 2,949,648 |
| 19 |  | Prospective study/ | 1,649,980 |
| 20 |  | Randomized controlled trials/ | 460,214 |
| 21 |  | 19 not 20 | 1,630,158 |
| 22 |  | Cohort analysis/ | 1,589,755 |
| 23 |  | Case control.tw. | 387,250 |
| 24 |  | (Case control adj (study or studies)).tw. | 319,863 |
| 25 |  | ((test-negative adj2 (study or studies or design?)) or (negative adj2 case-control)).tw. | 2,018 |
| 26 |  | (screening adj2 analys?s).tw. | 11,025 |
| 27 |  | (cohort adj (study or studies)).tw. | 906,158 |
| 28 |  | Cohort analy$.tw. | 36,195 |
| 29 |  | (Follow up adj (study or studies)).tw. | 136,847 |
| 30 |  | (observational adj (study or studies)).tw. | 479,717 |
| 31 |  | Longitudinal.tw. | 843,759 |
| 32 |  | Retrospective.tw. | 2,260,860 |
| 33 |  | Cross sectional.tw. | 1,359,734 |
| 34 |  | (Cohort adj (study or studies)).mp. | 1,137,574 |
| 35 |  | (epidemiologic$ adj (study or studies)).tw. | 227,846 |
| 36 |  | (cross sectional adj (study or studies)).tw. | 698,668 |
| 37 |  | (ecological adj2 (study or studies)).tw. | 18,932 |
| 38 |  | (((real-world or real-life) adj2 (stud* or data or evidence? or regist*)) or RWE or RWD).mp. | 104,527 |
| 39 |  | registries/ or regist*.mp. | 1,549,476 |
| 40 |  | or/10-18,21-39 | 10,741,253 |
| 41 |  | ((meta adj analy$) or metaanalys$ or meta analy$).tw. | 732,165 |
| 42 |  | ((systematic adj2 (review? or overview?)) or slr).tw. | 814,744 |
| 43 |  | or/41-42 | 1,148,333 |
| 44 |  | 40 or 43 | 11,509,792 |
| 45 | Outcomes (O) | (incidence adj2 model*).tw. | 3,512 |
| 46 |  | vaccine efficacy/ or (vaccin* adj2 (effectiveness or efficacy)).tw. | 112,724 |
| 47 |  | (vaccin* adj2 impact*).tw. | 8,842 |
| 48 |  | (protect* adj2 (duration or period or span)).tw. | 4,234 |
| 49 |  | (antigenicit* or immunogenicit*).tw. | 147,079 |
| 50 |  | (human serum bactericidal activity or hSBA).tw. | 459 |
| 51 |  | (antibod* persist* or immunity or immune response? or immun?persistence or immun?-persistence).tw. | 1,194,524 |
| 52 |  | ((duration or period) adj2 (response or immunity)).tw. | 37,308 |
| 53 |  | or/45-52 | 1,418,045 |
| 54 | S OR O | 44 or 53 | 12,779,840 |
| 55 | I+(S OR O) | 9 and 54 | 10,161 |
| 56 | Deduplication | limit 55 to yr="2013 -Current" | 5,378 |
| 57 |  | 55 not 56 | 4,783 |
| 58 |  | remove duplicates from 56 | 3,869 |
| 59 |  | remove duplicates from 57 | 3,541 |
| 60 |  | 58 or 59 | 7,410 |
| 61 | Time limit/Final | limit 60 to last 10 years | 3,578 |

IMD, invasive meningococcal disease; MenB, Meningitis B; PICOS, Population Intervention Comparator Outcome Study design; RWD, real-word data; RWE, real-world evidence

## Supplementary Table 2. Data extraction

| Publication details | Publication characteristics e.g., type of publication, journal, title, trial name, year etc. |
| --- | --- |
| Study details trial characteristics | E.g., study name, setting, duration, country, etc., study design, vaccination status (complete vaccination, partial vaccination, unvaccinated, other), population details (study population, inclusion/exclusion criteria, subgroups) |
| Baseline characteristics | - vaccination status (vaccine, scheme, duration, number of vaccine doses received, outbreak setting (e.g., related to the National Immunization Program (NIP) or private market uptake: outbreak setting helps to contextualize how vaccination efforts, whether through public health initiatives or private market participation, may contribute to or help control the spread of infectious diseases during an outbreak), baseline titers evaluation, the epidemiology of circulating strains, and results using, e.g., Multi-Antigenic Typing System (MATS) and Geometric Mean Antibody Titers (gMATs) to assess the immune response to the administered vaccines)) - general characteristics (number of patients, population details (e.g., general population, university students), behavioral characteristics (e.g., smoking status, attendance at nightclubs, attendance at mass gatherings), living arrangements (e.g., military barracks, student halls), population sub-groups, gender/sex, age, race/ethnicity, economic status, presence of chronic/non-chronic conditions, vaccination status, antibiotic use, medications exposure to cigarette smoke, education level) |
| Results | - vaccine impact against IMD (e.g., incidence rates, rate ratios, odds ratios, hazard ratios or percent differences to compare outcomes before and after the implementation of a meningococcal vaccine program or campaign) - vaccine effectiveness against IMD (vaccine effectiveness defined e.g. as the prevalence of IMD during vaccinated periods/of vaccinated cohort to the prevalence of IMD during unvaccinated periods/of unvaccinated cohort) - immune-persistence (e.g., antibody persistence, antibody response, immunity/immune response, duration/period of response/immunity) - immunogenicity (e.g., vaccine immunogenicity, antigenicity, serum bactericidal activity, human serum bactericidal activity (hSBA)) - duration of protection against IMD |
| Comments, studies’ limitations, additional notes. | |

IMD, invasive meningococcal disease

## Supplementary Table 3. Quality assessment of studies included in the meta-analysis, Newcastle-Ottawa Scale

| **Case-control studies** | | | | | | | | | |
| --- | --- | --- | --- | --- | --- | --- | --- | --- | --- |
| **Study** | **Selection** | | | | **Comparability** | **Exposure** | | | **Total** |
|  | **Q1** | **Q2** | **Q3** | **Q4** | **Q1** | **Q1** | **Q2** | **Q3** |  |
| Wang 2023^1^  Australia | 1 | 1 | 1 | 1 | 2 | 1 | 1 | 0 | 8 |
| Castilla 2023^2^  Spain | 1 | 1 | 1 | 1 | 2 | 1 | 1 | 0 | 8 |
| Lodi 2023^3^  Italy | 1 | 1 | 1 | 1 | 1 | 1 | 1 | 0 | 7 |
| Rodrigues 2020^4^  Portugal | 1 | 1 | 1 | 1 | 1 | 1 | 1 | 1 | 8 |
| **Cohort studies** |  |  |  |  |  |  |  |  |  |
| **Study** | **Selection** | | | | **Comparability** | **Exposure** | | | **Total** |
|  | **Q1** | **Q2** | **Q3** | **Q4** | **Q1** | **Q1** | **Q2** | **Q3** |  |
| Argante 2021^5^  England | 1 | 1 | 1 | 1 | 1 | 1 | 1 | 1 | 8 |
| Rodrigues 2023^6^ ^a^  Scotland | 1 | 1 | 1 | 1 | 1 | 1 | 1 | 1 | 8 |

^a^ Used in sensitivity analysis only

A study can be awarded a maximum of one star for each numbered item within the Selection and Exposure categories. A maximum of two stars can be given for Comparability. Ratings defined as high (7-8 stars), moderate (4-6 stars), or low (<4 stars).

**Newcastle-Ottawa Scale items for case-control studies**

| Domain | Item | Support for judgement | Star Eligibility & Assessment Rules |
| --- | --- | --- | --- |
| Selection | Q1 | Is the case definition adequate? | ⭐ for answer “a”, i.e., when study employs independent validation (e.g. >1 person/record/time/process to extract information, or reference to primary record source such as x-rays or medical/hospital records). Note: record linkage (e.g. ICD codes in database) or self-report with no reference to primary record classify as “b”. |
|  |  | a) ⭐ yes, with independent validation |  |
|  |  | b) yes, e.g. record linkage or based on self-reports |  |
|  |  | c) no description |  |
|  | Q2 | Representativeness of the cases | ⭐ for answer “a”, i.e., when study includes all eligible cases with outcome of interest over a defined period of time, all cases in a defined catchment area, all cases in a defined hospital or clinic, group of hospitals, health maintenance organisation, or an appropriate sample of those cases (e.g. random sample). Note: not satisfying requirements in part (a) classify as “b”. |
|  |  | a) ⭐ consecutive or obviously representative series of cases |  |
|  |  | b) potential for selection biases or not stated |  |
|  | Q3 | Selection of Controls | This item assesses whether the control series used in the study is derived from the same population as the cases and essentially would have been cases had the outcome been present.  ⭐ for answer “a” if study matches the answer “a”, i.e., includes community controls (i.e. same community as cases and would be cases if had outcome). Note: Hospital controls, within same community as cases (i.e. not another city) but derived from a hospitalized population classify as “b”. |
|  |  | a) ⭐ community controls |  |
|  |  | b) hospital controls |  |
|  |  | c) no description |  |
|  | Q4 | Definition of Controls | ⭐ for answer “a”, i.e., if cases are first occurrence of outcome, then it must explicitly state that controls have no history of this outcome. If cases have new (not necessarily first) occurrence of outcome, then controls with previous occurrences of outcome of interest should not be excluded. |
|  |  | a) ⭐ no history of disease (endpoint) |  |
|  |  | b) no description of source |  |
| Comparability | Q1 | Comparability of cases and controls on the basis of the design or analysis | Either cases and controls must be matched in the design and/or confounders must be adjusted for in the analysis. Statements of no differences between groups or that differences were not statistically significant are not sufficient for establishing comparability. Note: If the odds ratio for the exposure of interest is adjusted for the confounders listed, then the groups will be considered to be comparable on each variable used in the adjustment.  There may be multiple ratings for this item for different categories of exposure (e.g. ever vs. never, current vs. previous or never).  ⭐ for answer “a” or “b” - if both are matched, two stars are awarded (exception among domains). A maximum of 2 stars can be allotted in this category. |
|  |  | a) ⭐ study controls for the most important factor |  |
|  |  | b) ⭐ study controls for any additional factor |  |
| Exposure | Q1 | Ascertainment of exposure | ⭐ for answer “a”, no further requirements provided by the scale manuals. |
|  |  | a) ⭐ secure record (e.g. surgical records) |  |
|  |  | b) structured interview where blind to case/control status |  |
|  |  | c) interview not blinded to case/control status |  |
|  |  | d) written self-report or medical record only |  |
|  |  | e) no description |  |
|  | Q2 | Same method of ascertainment for cases and controls | ⭐ for answer “a”, no further requirements provided by the scale manuals. |
|  |  | a) ⭐ yes |  |
|  |  | b) no |  |
|  | Q3 | Non-Response rate | ⭐ for answer “a”, no further requirements provided by the scale manuals. |
|  |  | a) ⭐ same rate for both groups |  |
|  |  | b) non respondents described |  |
|  |  | c) rate different and no designation |  |

**Newcastle-Ottawa Scale items for cohort studies**

| Domain | Item | Support for judgement | Star Eligibility & Assessment Rules |
| --- | --- | --- | --- |
| Selection | Q1 | Representativeness of the exposed cohort | Item is assessing the representativeness of exposed individuals in the community, not the representativeness of the sample of women from some general population. For example, subjects derived from groups likely to contain middle class, better educated, health oriented women are likely to be representative of postmenopausal estrogen users while they are not representative of all women (e.g. members of a health maintenance organization (HMO) will be a representative sample of estrogen users. While the HMO may have an under-representation of ethnic groups, the poor, and poorly educated, these excluded groups are not the predominant users of estrogen).  ⭐ for answer “a” or “b”. |
|  |  | a) ⭐ truly representative of the average index in the community |  |
|  |  | b) ⭐ somewhat representative of the average index in the community |  |
|  |  | c) selected group of users e.g. nurses, volunteers |  |
|  |  | d) no description of the derivation of the cohort |  |
|  | Q2 | Selection of the non-exposed cohort | ⭐ for answer “a”, no further requirements provided by the scale manuals. |
|  |  | a) ⭐ drawn from the same community as the exposed cohort |  |
|  |  | b) drawn from a different source |  |
|  |  | c) no description of the derivation of the non-exposed cohort |  |
|  | Q3 | Ascertainment of exposure | ⭐ for answer “a” or “b”, no further requirements provided by the scale manuals. |
|  |  | a) ⭐ secure record (e.g. surgical records) |  |
|  |  | b) ⭐ structured interview |  |
|  |  | c) written self-report |  |
|  |  | d) no description |  |
|  | Q4 | Demonstration that outcome of interest was not present at start of study | In the case of mortality studies, outcome of interest is still the presence of a disease/ incident, rather than death. That is to say that a statement of no history of disease or incident earns a star.  ⭐ for answer “a”. |
|  |  | a) yes |  |
|  |  | b) no |  |
| Comparability | Q1 | Comparability of cohorts on the basis of the design or analysis | Either exposed and non-exposed individuals must be matched in the design and/or confounders must be adjusted for in the analysis. Statements of no differences between groups or that differences were not statistically significant are not sufficient for establishing comparability. Note: If the relative risk for the exposure of interest is adjusted for the confounders listed, then the groups will be considered to be comparable on each variable used in the adjustment.  There may be multiple ratings for this item for different categories of exposure (e.g. ever vs. never, current vs. previous or never)  ⭐ for answer “a” or “b” - if both are matched, two stars are awarded (exception among domains). A maximum of 2 stars can be allotted in this category. |
|  |  | a) ⭐ study controls for the most important factor |  |
|  |  | b) ⭐ study controls for any additional factor |  |
| Outcome | Q1 | Assessment of outcome | For some outcomes (e.g. fractured hip), reference to the medical record is sufficient to satisfy the requirement for confirmation of the fracture. This would not be adequate for vertebral fracture outcomes where reference to x-rays would be required.  ⭐ for answer “a” or “b”, if, for “a” independent or blind assessment is stated in the paper, or confirmation of the outcome by reference to secure records (x-rays, medical records, etc.) or, for “b” study mentions record linkage (e.g. identified through ICD codes on database records). Note: self-report (i.e. no reference to original medical records or x-rays to confirm the outcome) classify as “c”. |
|  |  | a) ⭐ independent blind assessment |  |
|  |  | b) ⭐ record linkage |  |
|  |  | c) self-report |  |
|  |  | d) no description |  |
|  | Q2 | Was follow-up long enough for outcomes to occur | An acceptable length of time should be decided before quality assessment begins (e.g. 5 yrs. for exposure to breast implants).  ⭐ for answer “a”. |
|  |  | a) ⭐ yes (an adequate follow up period for outcome of interest) |  |
|  |  | b) no |  |
|  | Q3 | Adequacy of follow up of cohorts | This item assesses the follow-up of the exposed and non-exposed cohorts to ensure that losses are not related to either the exposure or the outcome.  ⭐ for answer “a” or “b”. |
|  |  | a) ⭐ complete follow up - all subjects accounted for |  |
|  |  | b) ⭐ subjects lost to follow up unlikely to introduce bias - small number lost (follow up, or description provided of those lost) |  |
|  |  | c) adequate % of follow up rate (no description of those lost) |  |
|  |  | d) no statement |  |

## Supplementary Data 1. Calculation of VE estimates where not directly reported in source publication

Excel file describing the calculation of VE estimates for the study in Scotland^6^, using the screening method^7^ supplemented with data on vaccine coverage retrieved from Public Health Scotland reports^8^.

## Supplementary Figure 1. Scenario analyses


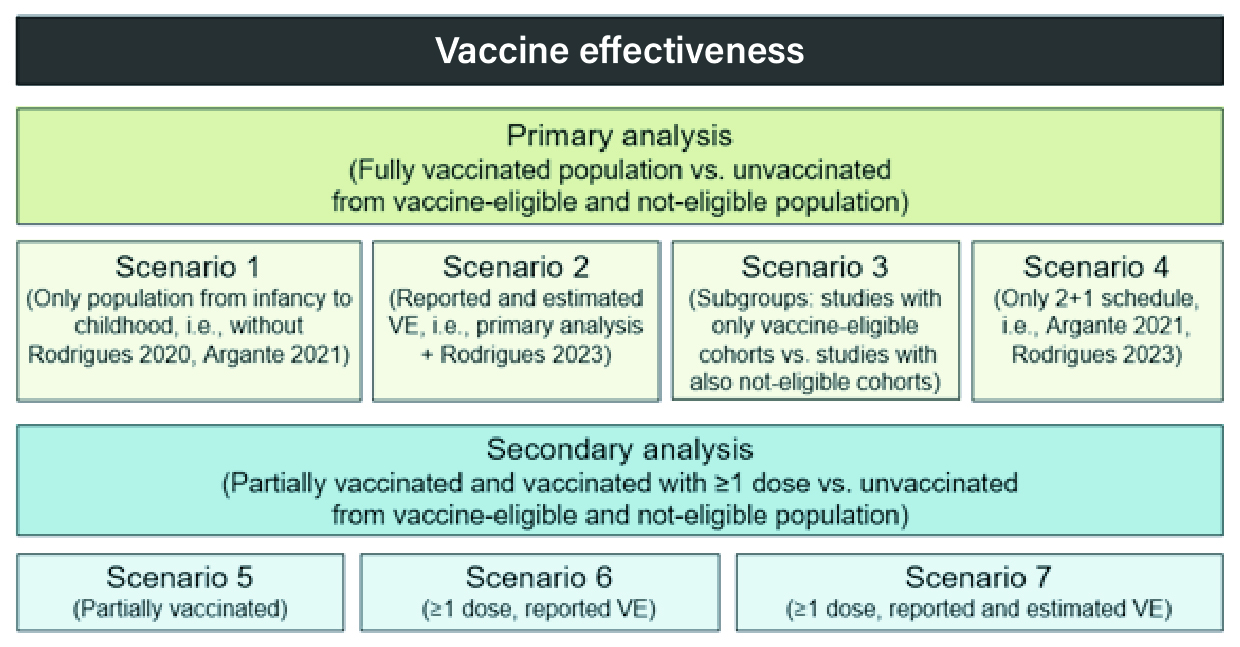


VE, vaccine effectiveness

Argante 2021^5^; Rodrigues 2020^4^; Rodrigues 2023^6^

## Supplementary Table 4. Results for vaccine impact against IMD caused by serogroup B

| **Study name** | **Study type/study period/Vaccine** | **Population** | **Vaccination status** | | **Parameter (95% CI)** | **Value** | | **p-value** |
| --- | --- | --- | --- | --- | --- | --- | --- | --- |
| **AUSTRALIA** | | | | | | | | |
| Wang 2023^1^* | • Case-control  • 2012–2021  • 4CMenB | **Infants** 12 weeks–<1 year^b^ | Overall^a^ | | Adjusted IRR | 0.369 (0.191–0.710) | | 0.003 |
|  |  |  |  |  | IRR | 0.401 (0.270–0.596) | | <0.001 |
|  |  | **Infants** 1 year of age^b^ | Overall^a^ | | Adjusted IRR | 0.487 (0.072–3.298) | | 0.461 |
|  |  |  |  |  | IRR | 0.530 (0.088–3.172) | | 0.487 |
|  |  | **Children** 2 years of age^b^ | Overall^a^ | | Adjusted IRR | 0.468 (0.082–2.662) | | 0.392 |
|  |  |  |  |  | IRR | 0.509 (0.095–2.734) | | 0.431 |
|  |  | **Children** 3 years of age^b^ | Overall^a^ | | IRR | 0 (Not calculable) | | NA |
|  |  | **Children** 4–6 years of age^b^ | Overall^a^ | | Adjusted IRR | 1.195 (0.172–8.289) | | 0.857 |
|  |  |  |  |  | IRR | 1.299 (0.195–8.673) | | 0.787 |
|  |  | **Adolescents** 15–18 years^c^ | Overall^a^ | | Adjusted IRR | 0.215 (0.069–0.670) | | 0.008 |
|  |  |  |  |  | IRR | 0.256 (0.091–0.722) | | 0.010 |
|  |  | **Adolescents** 19–21 years^c^ | Overall^a^ | | Adjusted IRR | 0.445 (0.136–1.459) | | 0.181 |
|  |  |  |  |  | IRR | 0.530 (0.187–1.496) | | 0.230 |
|  |  | **Adolescents** 22–25 years^c^ | Overall^a^ | | Adjusted IRR | 0.224 (0.039–1.306) | | 0.096 |
|  |  |  |  |  | IRR | 0.267 (0.048–1.475) | | 0.130 |
| Wang 2022^9^* | • Cohort, case-control  • 2011–2019  • 4CMenB | **Infants** 12 weeks–11 months of age^d^ | Overall^a^ | | Adjusted IRR | 0.40 (0.23–0.69) | | 0.0011 |
|  |  |  |  |  | IRR | 0.40 (0.27–0.59) | | <0.0001 |
|  |  | **Infants** 1 year of age^d^ | Overall^a^ | | Adjusted IRR | 0.79 (0.16–3.87) | | 0.77 |
|  |  |  |  |  | IRR | 0.78 (0.16–3.89) | | 0.77 |
|  |  | **Children** 2 years of age^d^ | Overall^a^ | | Adjusted IRR | 0.75 (0.18–3.14) | | 0.70 |
|  |  |  |  |  | IRR | 0.75 (0.17–3.24) | | 0.70 |
|  |  | **Children** 3 years of age^d^ | Overall^a^ | | Adjusted IRR | Not calculable | | NA |
|  |  |  |  |  | IRR | Not calculable | | NA |
|  |  | **Infants** 12 weeks–<1 year of age^e^ | Overall^a^ | | Adjusted IRR | 0.42 (0.26–0.68) | | 0.0005 |
|  |  |  |  | | IRR | 0.40 (0.29–0.55) | | <0.0001 |
|  |  | **Children** 4 years of age^d^ | Overall^a^ | | Adjusted IRR | 3.00 (0.47–18.79) | | 0.24 |
|  |  |  |  |  | IRR | 2.98 (0.44–20.05) | | 0.26 |
|  |  | **Children** 1–4 years of age^e^ | Overall^a^ | | Adjusted IRR | Year 1 (February 2019–January 2020): 1.20 (0.73–1.99) | | 0.47 |
|  |  |  |  |  | IRR | Year 1 (February 2019–January 2020): 1.04 (0.77–1.40)  Year 2 (February 2020–January 2021): Not calculable | | Year 1 (February 2019–January 2020): 0.8 |
|  |  | **Children** 5 years of age^d^ | Overall^a^ | | Adjusted IRR | Not calculable | | NA |
|  |  |  |  |  | IRR | Not calculable | | NA |
|  |  | **Adolescents** 15–18 years of age^e^ | Overall^a^ | | Adjusted IRR | 0.27 (0.06–1.16) | | 0.078 |
|  |  |  |  |  | IRR | 0.26 (0.06–1.15) | | 0.08 |
|  |  | **Adolescents** 19–21 years of age^e^ | Overall^a^ | | IRR | Year 1 (February 2019–January 2020): 1.04 (0.70–1.54)  Year 2 (February 2020–January 2021): Not calculable | | Year 1 (February 2019–January 2020): 0.86 |
|  |  |  |  |  | Adjusted IRR | Year 1 (February 2019–January 2020): 1.20 (0.70–2.06) | | 0.51 |
| **EUROPE** | | | | | | | | |
| Ladhani 2020^10^ | • Cohort  • 2015–2018  • 4CMenB | **Infants** 0–8 weeks of age (2015–2016 vs. 2010–2015) | Overall^a^ | | IRR | 0.52 (0.24–1.14) | | NR |
|  |  | **Infants** 0–8 weeks of age (2016–2017 vs. 2010–2015) | Overall^a^ | | IRR | 0.60 (0.28–1.25) | | NR |
|  |  | **Infants** 0–8 weeks of age (2017–2018 vs. 2010–2015) | Overall^a^ | | IRR | 0.75 (0.38–1.48) | | NR |
|  |  | **Infants** 9–17 weeks of age (2015–2016 vs. 2010–2015) | Overall^a^ | | IRR | 0.55 (0.29–1.02) | | NR |
|  |  | **Infants** 9–17 weeks of age (2016–2017 vs. 2010–2015) | Overall^a^ | | IRR | 1.05 (0.65–1.69) | | NR |
|  |  | **Infants** 9–17 weeks of age (2017–2018 vs. 2010–2015) | Overall^a^ | | IRR | 1.01 (0.62–1.65) | | NR |
|  |  | **Infants** 18–51 weeks of age (2015–2016 vs. 2010–2015) | Overall^a^ | | IRR | 0.63 (0.48–0.83) | | NR |
|  |  | **Infants** 18–51 weeks of age (2016–2017 vs. 2010–2015) | Overall^a^ | | IRR | 0.23 (0.15–0.35) | | NR |
|  |  | **Infants** 18–51 weeks of age (2017–2018 vs. 2010–2015) | Overall^a^ | | IRR | 0.31 (0.21–0.45) | | NR |
|  |  | **Children** 1 year of age (2015–2016 vs. 2010–2015) | Overall^a^ | | IRR | 0.83 (0.63–1.11) | | NR |
|  |  | **Children** 1 year of age (2016–2017 vs. 2010–2015) | Overall^a^ | | IRR | 0.42 (0.28–0.61) | | NR |
|  |  | **Children** 1 year of age (2017–2018 vs. 2010–2015) | Overall^a^ | | IRR | 0.20 (0.12–0.35) | | NR |
|  |  | **Children** 2 years of age (2015–2016 vs. 2010–2015) | Overall^a^ | | IRR | 1.02 (0.73–1.43) | | NR |
|  |  | **Children** 2 years of age (2016–2017 vs. 2010–2015) | Overall^a^ | | IRR | 1.18 (0.85–1.62) | | NR |
|  |  | **Children** 2 years of age (2017–2018 vs. 2010–2015) | Overall^a^ | | IRR | 0.46 (0.29–0.75) | | NR |
|  |  | **Children** 3 years of age (2015–-2016 vs. 2010–2015) | Overall^a^ | | IRR | 0.83 (0.52–1.33) | | NR |
|  |  | **Children** 3 years of age (2016–2017 vs. 2010–2015) | Overall^a^ | | IRR | 0.97 (0.62–1.52) | | NR |
|  |  | **Children** 3 years of age (2017–2018 vs. 2010–2015) | Overall^a^ | | IRR | 1.11 (0.72–1.70) | | NR |
|  |  | **Children** 4 years of age (2015–2016 vs. 2010–2015) | Overall^a^ | | IRR | 1.04 (0.65–1.66) | | NR |
|  |  | **Children** 4 years of age (2016–2017 vs. 2010–2015) | Overall^a^ | | IRR | 0.90 (0.55–1.48) | | NR |
|  |  | **Children** 4 years of age (2017–2018 vs. 2010–2015) | Overall^a^ | | IRR | 1.02 (0.63–1.64) | | NR |
|  |  |  |  | |  |  | |  |
| Argante 2021^5^** | • Incidence modelling based on cohort  • 2011–2018  • 4CMenB | **Infants**, **children** | 1 dose | | Adjusted IRR | 0.66 (0.50–0.88) | | <0.001 |
|  |  |  | 2 doses | | Adjusted IRR | 0.21 (0.15–0.28) | | <0.001 |
|  |  |  | 3 doses | | Adjusted IRR | 0.19 (0.12–0.29) | | <0.001 |
|  |  |  | 1 dose vs. 2 doses | | Adjusted IRR | 3.13 (2.11–4.62) | | NR |
|  |  | **Infants**, **children** in (2011–2012) | Unvaccinated | | Adjusted IRR | 1.15 (1.06–1.25) | | <0.001 |
|  |  | **Infants**, **children** (2012–2013) | Unvaccinated | | Adjusted IRR | 1.10 (1.01–1.19) | | <0.001 |
|  |  | **Infants**, **children** (2013–2014) | Unvaccinated | | Adjusted IRR | 0.83 (0.76–0.91) | | <0.001 |
|  |  | **Infants**, **children** (2014–2015) | Unvaccinated | | Adjusted IRR | 0.85 (0.78–0.93) | | <0.001 |
|  |  | **Infants**, **children** (2015–2016) | Unvaccinated | | Adjusted IRR | 0.98 (0.91–1.07) | | <0.001 |
|  |  | **Infants**, **children** (2016–2017) | Unvaccinated | | Adjusted IRR | 1.03 (0.94–1.12) | | <0.001 |
|  |  | **Infants**, **children** (2017–2018) | Unvaccinated | | Adjusted IRR | 1.06 (0.97–1.16) | | <0.001 |
| Azzari 2020^11^ | • Cohort  • 2006–2018  • 4CMenB | **Infants** 0–1 year of age (Tuscany) | Post vaccination period (vaccinated cases) | | IRR | 0.131 (NR) | | NR |
|  |  |  | Post vaccination cohort | | IRR | 0.260 (NR) | | NR |
|  |  |  | Post vaccination cohort | | Overall vaccine impact^f^ | 74%  (-15–94) | | NR |
|  |  |  |  |  | Total impact^g^ | 87% (NR) | | NR |
|  |  | **Children** 1–2 years of age (Tuscany) | Post vaccination cohort | | Overall vaccine impact^f^ | 49% (NR) | | NR |
|  |  |  |  |  | IRR | 0.507 (NR) | | NR |
|  |  | **Children** 2–3 years of age (Tuscany) | Post vaccination cohort | | IRR | 1.838 (NR) | | NR |
|  |  |  |  |  |  |  |  |  |
|  |  | **Infants and children** 0–5 years of age (Tuscany) | Post vaccination cohort | | Overall vaccine impact^f^ | 68%  (9–88) | | NR |
|  |  |  |  |  | Total impact^g^ | 94% (56–99) | | NR |
|  |  |  |  |  | IRR | 0.320 (NR) | | NR |
|  |  |  | Post vaccination period (vaccinated cases) | | IRR | 0.060 (NR) | | NR |
|  |  | **Infants** 0–1 year of age (Veneto) | Post vaccination cohort | | Overall vaccine impact^f^ | 58%  (-43–87) | | NR |
|  |  |  |  |  | IRR | 0.421 (NR) | | NR |
|  |  | **Children** 1–2 years of age (Veneto) | Post vaccination cohort (vaccinated cases) | | IRR | 0.590 (NR) | | NR |
|  |  |  | Post vaccination cohort | | IRR | 1.764 (NR) | | NR |
|  |  |  |  |  | Total impact^g^ | 41% (NR) | | NR |
|  |  | **Children** 2–3 years of age (Veneto) | Post vaccination cohort | | IRR | 1.158 (NR) | | NR |
|  |  | **Infants and children** 0–4 years of age (Veneto) | Post vaccination cohort | | Overall vaccine impact^f^ | 31%  (-56–69) | | NR |
|  |  |  |  |  | Total impact^g^ | 90% (57–97) | | NR |
|  |  |  |  |  | IRR | 0.692 (NR) | | NR |
|  |  |  | Post vaccination cohort (vaccinated cases) | | IRR | 0.103 (NR) | | NR |
| **EUROPE** | | | | | | | | |
| Ladhani 2020^10^** | • Cohort  • 2015–2018  • 4CMenB | **Infants** 9–17 weeks of age (2015–2016 vs. 2010–2015) | Overall^a^ | | Adjusted IRR | 0.62 (0.32–1.20) | | NR |
|  |  | **Infants** 9–17 weeks of age (2016–2017 vs. 2010–2015) | Overall^a^ | | Adjusted IRR | 1.07 (0.63–1.81) | | NR |
|  |  | **Infants** 9–17 weeks of age (2017–2018 vs. 2010–2015) | Overall^a^ | | Adjusted IRR | 0.99 (0.56–1.74) | | NR |
|  |  | **Infants** 18–51 weeks of age (2015–2016 vs. 2010–2015) | Overall^a^ | | Adjusted IRR | 0.72 (0.52–0.99) | | NR |
|  |  | **Infants** 18–51 weeks of age (2016–2017 vs. 2010–2015) | Overall^a^ | | Adjusted IRR | 0.23 (0.14–0.38) | | NR |
|  |  | **Infants** 18–51 weeks of age (2017–2018 vs. 2010–2015) | Overall^a^ | | Adjusted IRR | 0.30 (0.19–0.49) | | NR |
|  |  | **Children** 1 year of age (2016–2017 vs. 2010–2015) | Overall^a^ | | Adjusted IRR | 0.43 (0.28–0.66) | | NR |
|  |  | **Children** 1 year of age (2017–2018 vs. 2010–2015) | Overall^a^ | | Adjusted IRR | 0.20 (0.11–0.36) | | NR |
|  |  | **Children** 2 years of age (2017–2018 vs. 2010–2015) | Overall^a^ | | Adjusted IRR | 0.43 (0.25–0.74) | | NR |
|  |  | **Infants and children** <5 years of age | Vaccine-eligible cohort | | IRR | 0.25 (0.19–0.36) | | NR |
|  |  |  |  |  | Reduction in IMD incidence | 75% (NR) | | NR |
|  |  | **Infants and children** <5 years of age | Vaccine selective cohort | | IRR | 0.55 (0.43–0.70) | | NR |
|  |  |  |  |  | Reduction in IMD incidence | 45% (NR) | | NR |
|  |  | **Infants** 9–17 weeks of age (2015–2016 vs. 2010–2015) | Overall^a^ | | Adjusted IRR | 0.62 (0.32–1.20) | | NR |
|  |  | **Infants** 9–17 weeks of age (2016–2017 vs. 2010–2015) | Overall^a^ | | Adjusted IRR | 1.07 (0.63–1.81) | | NR |
|  |  | **Infants** 9–17 weeks of age (2017–2018 vs. 2010–2015) | Overall^a^ | | Adjusted IRR | 0.99 (0.56–1.74) | | NR |
|  |  | **Infants** 18–51 weeks of age (2015–2016 vs. 2010–2015) | Overall^a^ | | Adjusted IRR | 0.72 (0.52–0.99) | | NR |
|  |  | **Infants** 18–51 weeks of age (2016–2017 vs. 2010–2015) | Overall^a^ | | Adjusted IRR | 0.23 (0.14–0.38) | | NR |
|  |  | **Infants** 18–51 weeks of age (2017–2018 vs. 2010–2015) | Overall^a^ | | Adjusted IRR | 0.30 (0.19–0.49) | | NR |
|  |  | **Children** 1 year of age (2016–2017 vs. 2010–2015) | Overall^a^ | | Adjusted IRR | 0.43 (0.28–0.66) | | NR |
|  |  | **Children** 1 year of age (2017–2018 vs. 2010–2015) | Overall^a^ | | Adjusted IRR | 0.20 (0.11–0.36) | | NR |
|  |  | **Children** 2 years of age (2017–2018 vs. 2010–2015) | Overall^a^ | | Adjusted IRR | 0.43 (0.25–0.74) | | NR |
|  |  | **Infants and children** <5 years of age | Vaccine-eligible cohort | | IRR | 0.25 (0.19–0.36) | | NR |
|  |  |  |  | | Reduction in IMD incidence | 75% (NR) | | NR |
|  |  | **Infants and children** <5 years of age | Vaccine selective cohort | | IRR | 0.55 (0.43–0.70) | | NR |
|  |  |  |  | | Reduction in IMD incidence | 45% (NR) | | NR |
| **EUROPE** | | | | | | | | |
| Lodi 2023^3^ | • Cohort  • 2006–2020  • 4CMenB | **Infants** **and** **children** <6 years of age (Tuscany, Veneto, Piedmont) | | Overall^a^ | RCR | 48% (31–64) | NR | |
|  |  |  |  | Vaccinated | RCR | 90% (75–97) | NR | |
|  |  |  |  | Overall^a^ | Reduction in IRR per 100,000 | 0.50 (0.14–0.71) | NR | |
|  |  |  |  | Vaccinated | Reduction in IRR per 100,000 | 0.89 (0.64–0.96) | NR | |
|  |  | **Infants** **and children** <6 years of age in Tuscany | | Overall^a^ | RCR | 72% (46–89) | NR | |
|  |  |  |  | Vaccinated | RCR | 93% (69–99) | NR | |
|  |  |  |  | Overall^a^ | IRR per 100,000 | 0.26 (0.09–0.74) | 0.006 | |
|  |  |  |  | Overall^a^ | Reduction in IRR per 100,000 | 0.74 (0.26–0.91) | NR | |
|  |  |  |  | Vaccinated | Reduction in IRR per 100,000 | 0.95 (0.63–0.99) | NR | |
|  |  | **Infants** **and children** <6 years of age in Veneto | | Overall^a^ | RCR | 37% (17–64) | NR | |
|  |  |  |  | Vaccinated | RCR | 84% (70–96) | NR | |
|  |  |  |  | Overall^a^ | Reduction in IRR per 100,000 | 0.45 (-0.18–0.74) | NR | |
|  |  |  |  | Vaccinated | Reduction in IRR per 100,000 | 0.83 (0.28–0.96) | NR | |
|  |  | **Infants** **and children** <6 years of age (Piedmont) | | Overall^a^ | RCR | 22% (1–69) | NR | |
|  |  |  |  |  | Reduction in IRR per 100,000 | 0.36 (-1.18–0.81) | NR | |
|  |  |  |  | Vaccinated | RCR | 100% (51–100) | NR | |
| **EUROPE** | | | | | | | | |
| Mensah 2023^12^ | • Cohort  • 2010–2020  • 4CMenB | **Infants** <1 year of age | | Overall^a^ | Reduction in IMD cases | -60% (NR) | NR | |
|  |  | **Children** 1–4 years of age | | Overall^a^ | Reduction in IMD cases | -50% (NR) | NR | |
|  |  | **Children** ≥5 years of age | | Overall^a^ | Reduction in IMD cases | -4% (NR) | NR | |
| Parikh 2016^13^** | • Cohort  • 2015–2016  • 4CMenB | **Infants**  For patients receiving the second dose ≥18 weeks of age  For patients receiving only the first dose 2–4 months (10–17 weeks) of age | | 2 doses | Adjusted IRR | 0.53 (0.33–0.87) | 0.012 | |
|  |  |  |  |  | Relative IRR | 0.62 (0.37–1.04) | 0.070 | |
|  |  |  |  | 1 dose | Adjusted IRR | 0.66 (0.34–-1.28) | 0.216 | |
|  |  |  |  |  | Relative IRR | 0.76 (0.38–1.52) | 0.439 | |
|  |  |  |  | Vaccine-eligible | Adjusted IRR | 0.50 (0.36–0.71) | 0.0001 | |
|  |  |  |  |  | Relative IRR | 0.58 (0.40–0.85) | 0.005 | |
|  |  | **Infants and children** <5 years with MenB disease and excluding vaccine-eligible and equivalent pre-vaccine cohorts | | Overall^a^ | Adjusted IRR | 0.86 (0.73–1.01) | 0.073 | |
|  |  | **Infants** born between May 1 and June 30, 2015 (Catch-up cohort) | | Overall^a^ | Adjusted IRR | 0.36 (0.18–0.72) | 0.004 | |
|  |  |  |  |  | Relative IRR | 0.42 (0.21–0.85) | 0.016 | |
|  | • Trend model  • 2015–2016  • 4CMenB | **Infants**  For patients receiving the second dose ≥18 weeks of age  For patients receiving only the first dose 2–4 months (10–17 weeks) of age | | 2 doses | Adjusted IRR | 0.68 (0.41–1.13) | 0.134 | |
|  |  |  |  | 1 dose | Adjusted IRR | 0.84 (0.43–1.65) | 0.606 | |
|  |  |  |  | Vaccine-eligible | Adjusted IRR | 0.64 (0.45–0.92) | 0.015 | |
|  |  | **Infants** born between May 1 and June 30, 2015 | | Overall^a^ | Adjusted IRR | 0.46 (0.23–0.93) | 0.029 | |
| **NORTH** **AMERICA** | | | | | | | | |
| Martinón-Torres 2021^14^***^ | • Cohort  • 2004–2019^  • 4CMenB | **Infants, children and adolescents**:  Residents ≤20 years of age in SLSJ region | | Post vaccination period | IRR | 0.06 (NR) | <0.06 | |
|  |  | **Adolescents/young** **adults**:  Residents >20 years of age in SLSJ region | | Post vaccination period | IRR | 0.33 (NR) | NR | |
| Deceuninck 2019^15^*** | • Cohort  • 2004–2018  • 4CMenB | **Infants, children, adolescents and young** **adults**:  All ages in province of Quebec | | Post vaccination period | IRR | 0.37 (NR) | <0.0001 | |
|  |  | **Infants, children, adolescents and young** **adults**:  All ages in SLSJ region | | Post vaccination period | IRR | 0.13 (NR) | <0.0001 | |
|  |  | **Infants, children, adolescents and young** **adults**:  All ages in other regions | | Post vaccination period | IRR | 0.42 (NR) | <0.0001 | |
|  |  | **Adolescents/young** **adults**:  Residents >20 years of age in province of Quebec | | Post vaccination period | IRR | 0.44 (NR) | 0.0001 | |
|  |  | **Adolescents/young** **adults**:  Residents >20 years of age in SLSJ region | | Post vaccination period | IRR | 0.41 (NR) | 0.1 | |
|  |  | **Adolescents/young** **adults**:  Residents >20 years of age in other regions | | Post vaccination period | IRR | 0.44 (NR) | <0.0001 | |
|  |  | **Infants, children and adolescents**:  Residents ≤20 years of age in province of Quebec | | Post vaccination period | IRR | 0.35 (NR) | <0.0001 | |
|  |  | **Infants, children and adolescents**:  Residents ≤20 years of age in SLSJ region | | Post vaccination period | IRR | 0.04 (NR) | 0.0013 | |
|  |  | **Infants, children and adolescents**:  Residents ≤20 years of age in other regions | | Post vaccination period | IRR | 0.42 (NR) | <0.0001 | |
| De Wals 2017^16^*** | •Cohort  • 2014–2016  • 4CMenB | **Infants, children, adolescents and young adults**:  All ages in province of Quebec | | Post vaccination period | IRR | 0.33 (NR) | <0.0001 | |
|  |  | **Infants, children, adolescents and young adults**:  All ages in SLSJ region | | Post vaccination period | IRR | 0.08 (NR) | <0.0001 | |
|  |  | **Infants, children, adolescents and young adults**:  All ages in other regions | | Post vaccination period | IRR | 0.47 (NR) | <0.0001 | |
|  |  | **Adolescents/young adults**:  Residents >20 years of age in province of Quebec | | Post vaccination period | IRR | 0.45 (NR) | 0.0001 | |
|  |  | **Adolescents/young adults**:  Residents >20 years of age in SLSJ region | | Post vaccination period | IRR | 0.33 (NR) | 0.13 | |
|  | | | | | | | | |
|  |  | **Adolescents/young** **adults**:  Residents >20 years of age in other regions | | Post vaccination period | IRR | 0.47 (NR) | <0.0001 | |
|  |  | **Infants, children and adolescents**:  Residents ≤20 years of age in province of Quebec | | Post vaccination period | IRR | 0.39 (NR) | <0.0001 | |
|  |  | **Infants, children and adolescents**:  Residents ≤20 years of age in SLSJ region | | Post vaccination period | IRR | 0.00 (NR) | <0.0001 | |
|  |  | **Infants, children and adolescents**:  Residents ≤20 years of age in other regions | | Post vaccination period | IRR | 0.49 (NR) | <.0001 | |
|  |  |  |  | |  |  | |  |

CI, confidence interval; IRR, incidence rate ratio; NA, not applicable; NR, not reported; RCR, relative case reduction; SLSJ, Saguenay-Lac-Saint-Jean

*Linked references.

**Linked references.

***Linked references.

^Personal communication with De Wals (data for 5-year post vaccination).

a) Including both vaccinated and unvaccinated cases.

b) Post vaccination period (2018–2021) vs. Pre-vaccination period (2012–2018).

c) Post vaccination period (2019–2022) vs. Pre-vaccination period (2011–2019).

d) Post vaccination (October, 2018–September, 2020) vs. Pre-vaccination period (October, 2012–September, 2018).

e) Post vaccination (February, 2019–January, 2021) vs. Pre-vaccination period (February, 2011–January, 2019).

f) Effect of the 4CMenB vaccination program on the entire population, regardless of vaccination status.

g) The effect of 4CMenB vaccination on the vaccinated population.

**References**

1 Wang, B. *et al.* 4CMenB sustained vaccine effectiveness against invasive meningococcal B disease and gonorrhoea at three years post programme implementation. *J Infect* **87**, 95-102 (2023). <https://doi.org:10.1016/j.jinf.2023.05.021>

2 Castilla, J. *et al.* Effectiveness of a Meningococcal Group B Vaccine (4CMenB) in Children. *N Engl J Med* **388**, 427-438 (2023). <https://doi.org:10.1056/NEJMoa2206433>

3 Lodi, L. *et al.* Four-Component Recombinant Protein-Based Vaccine Effectiveness Against Serogroup B Meningococcal Disease in Italy. *JAMA Netw Open* **6**, e2329678 (2023). <https://doi.org:10.1001/jamanetworkopen.2023.29678>

4 Rodrigues, F. M. P. *et al.* Association of Use of a Meningococcus Group B Vaccine With Group B Invasive Meningococcal Disease Among Children in Portugal. *JAMA* **324**, 2187-2194 (2020). <https://doi.org:10.1001/jama.2020.20449>

5 Argante, L., Abbing-Karahagopian, V., Vadivelu, K., Rappuoli, R. & Medini, D. A re-assessment of 4CMenB vaccine effectiveness against serogroup B invasive meningococcal disease in England based on an incidence model. *BMC Infect Dis* **21**, 1244 (2021). <https://doi.org:10.1186/s12879-021-06906-x>

6 Rodrigues, C. M. C. *et al.* Exploiting Real-Time Genomic Surveillance Data To Assess 4CMenB Meningococcal Vaccine Performance in Scotland, 2015 to 2022. *mBio* **14**, e0049923 (2023). <https://doi.org:10.1128/mbio.00499-23>

7 Farrington, C. P. Estimation of vaccine effectiveness using the screening method. *Int J Epidemiol* **22**, 742-746 (1993). <https://doi.org:10.1093/ije/22.4.742>

8 Public Health Scotland. *All releases of Childhood immunisation statistics Scotland*, <<https://publichealthscotland.scot/publications/show-all-releases?id=20562>> (2024).

9 Wang, B. *et al.* Effectiveness and impact of the 4CMenB vaccine against invasive serogroup B meningococcal disease and gonorrhoea in an infant, child, and adolescent programme: an observational cohort and case-control study. *Lancet Infect Dis* **22**, 1011-1020 (2022). <https://doi.org:10.1016/S1473-3099(21)00754-4>

10 Ladhani, S. N. *et al.* Vaccination of Infants with Meningococcal Group B Vaccine (4CMenB) in England. *N Engl J Med* **382**, 309-317 (2020). <https://doi.org:10.1056/NEJMoa1901229>

11 Azzari, C. *et al.* Effectiveness and Impact of the 4CMenB Vaccine against Group B Meningococcal Disease in Two Italian Regions Using Different Vaccination Schedules: A Five-Year Retrospective Observational Study (2014-2018). *Vaccines (Basel)* **8** (2020). <https://doi.org:10.3390/vaccines8030469>

12 Mensah, A. A. *et al.* Outcomes of meningococcal serogroup B disease in children after implementation of routine infant 4CMenB vaccination in England: an active, prospective, national surveillance study. *Lancet Child Adolesc Health* **7**, 190-198 (2023). <https://doi.org:10.1016/S2352-4642(22)00379-0>

13 Parikh, S. R. *et al.* Effectiveness and impact of a reduced infant schedule of 4CMenB vaccine against group B meningococcal disease in England: a national observational cohort study. *Lancet* **388**, 2775-2782 (2016). <https://doi.org:10.1016/S0140-6736(16)31921-3>

14 Martinón-Torres, F. *et al.* Recent advances in meningococcal B disease prevention: real-world evidence from 4CMenB vaccination. *J Infect* **83**, 17-26 (2021). <https://doi.org:10.1016/j.jinf.2021.04.031>

15 Deceuninck, G. *et al.* Impact of a mass vaccination campaign against Serogroup B meningococcal disease in the Saguenay-Lac-Saint-Jean region of Quebec four years after its launch. *Vaccine* **37**, 4243-4245 (2019). <https://doi.org:10.1016/j.vaccine.2019.06.021>

16 De Wals, P. *et al.* Impact of an Immunization Campaign to Control an Increased Incidence of Serogroup B Meningococcal Disease in One Region of Quebec, Canada. *Clin Infect Dis* **64**, 1263-1267 (2017). <https://doi.org:10.1093/cid/cix154>
