# Supplementary material for: Real-world effectiveness and impact of the 4CMenB vaccine against serogroup B invasive meningococcal disease: a systematic review and meta-analysis
Source: NPJ Vaccines. 2026 Jul 6;11:153. doi: 10.1038/s41541-026-01511-y (PMC13401604; doi:10.1038/s41541-026-01511-y)
Supplement: Supplementary file 2 — supplementary file: video script [file 41541_2026_1511_MOESM2_ESM.docx]

This video summarizes the findings from the paper titled: "Real-world effectiveness and impact of the 4CMenB vaccine against serogroup B invasive meningococcal disease: a systematic review and meta-analysis" by Dr. Marijic and co-authors.

Invasive meningococcal disease, or IMD, is a severe infection that can prove fatal within hours, and up to 20% of survivors may experience long-term disabilities. IMD occurs most frequently in infants and young children, with a second peak in adolescents. Serogroup B is the most common type in Europe, North America and Australasia.

Serogroup B-IMD can be prevented by the 4CMenB vaccine, which is authorized for infants, children and adults. Real-world data from over 10 years of use have supported clinical data obtained from early trials.

In this study, we searched systematically for studies reporting real-world evidence on 4CMenB impact and effectiveness in infants, children and adolescents, published in the last ten years across the world. We found 14 publications reporting real-world evidence on vaccine impact or effectiveness of 4CMenB.

These publications showed that 4CMenB vaccination programs were associated with a substantial decrease in serogroup B-IMD in infants/children/adolescents/young adults in Australia, UK, Italy and Canada.

Twelve publications reported evidence on vaccine effectiveness. Of these, five papers, from Australia, Italy, Portugal, Spain and UK, reported data on vaccine effectiveness of 4CMenB in fully vaccinated infants and children, and were similar enough to combine their results in a meta-analysis. This meta-analysis estimated overall 4CMenB effectiveness at approximately 80%.

In addition, a study from South Australia reported 4CMenB vaccine effectiveness of 83.5–100% in adolescents, showing strong protection in this age group.

Limitations of this study include Variation across studies;  Limited data in adolescents,

potential confounding in observational studies, and  limited representation from low- and middle-income countries.

In summary, this meta-analysis shows that 4CMenB provides strong protection against serogroup B-IMD in infants and children in real-world use across various high-income countries. This is the first meta-analysis to combine real-world effectiveness data for 4CMenB in infants and children.

**Word count excluding title paragraph: 333**
